# Protein phosphatase-1 regulates the binding of filamin C to FILIP1 in cultured skeletal muscle cells under mechanical stress

**DOI:** 10.1038/s41598-024-78953-8

**Published:** 2024-11-09

**Authors:** Thomas Kokot, Johannes P. Zimmermann, Anja N. Schwäble, Lena Reimann, Anna L. Herr, Nico Höfflin, Maja Köhn, Bettina Warscheid

**Affiliations:** 1 Integrative Signaling Research, Institute of Biology III, University of Freiburg, Freiburg, Germany; 2 Signalling Research Centres BIOSS and CIBSS, University of Freiburg, Freiburg, Germany; 3 Biochemistry II, Theodor-Boveri-Institut, Biozentrum, Faculty of Chemistry and Pharmacy, University of Würzburg, Würzburg, Germany; 4 Biochemistry - Functional Proteomics, Institute of Biology II, University of Freiburg, Freiburg, Germany; 5 Current address: Celonic AG, Basel, Switzerland; 6 Current address: Sartorius CellGenix GmbH, Freiburg, Germany

**Keywords:** Proteins, Phosphorylation, Stress signalling

## Abstract

The actin-binding protein filamin c (FLNc) is a key mediator in the response of skeletal muscle cells to mechanical stress. In addition to its function as a structural scaffold, FLNc acts as a signaling adaptor which is phosphorylated at S2234 in its mechanosensitive domain 20 (d20) through AKT. Here, we discovered a strong dephosphorylation of FLNc-pS2234 in cultured skeletal myotubes under acute mechanical stress, despite high AKT activity. We found that all three protein phosphatase 1 (PP1) isoforms are part of the FLNc d18-21 interactome. Enzymatic assays demonstrate that PP1 efficiently dephosphorylates FLNc-pS2234 and in vitro and in cells upon PP1 activation using specific modulators. FLNc-pS2234 dephosphorylation promotes the interaction with FILIP1, a mediator for filamin degradation. Altogether, we present a model in which dephosphorylation of FLNc d20 by the dominant action of PP1c prevails over AKT activity to promote the binding of the filamin degradation-inducing factor FILIP1 during acute mechanical stress.

**Supplementary Information:**

The online version contains supplementary material available at 10.1038/s41598-024-78953-8.

## Introduction

For living organisms, it is a fundamental requirement to endure and overcome various forms of cellular stress while maintaining a functional proteome and homeostasis^1–3^. Amongst various stressors, mechanical force has been more recently recognized as an important source of acute or chronic forms of cellular stress in multicellular organisms^1^. During physical activity, mechanical strain acts vigorously on cross-striated muscle cells, and especially on the mechanosensitive components of the myofibrillar Z-disc, which constitutes the boundary of adjacent sarcomeres^4–6^. Various forms of muscle diseases such as different variants of myopathies and cardiomyopathies that lead to progressive muscle weakness and heart failure are caused by aberrant forms of Z-disc and Z-disc-associated proteins^5–7^.

A key component associated with the myofibrillar Z-disc is the large actin-binding protein filamin C (FLNc). FLNc is mostly expressed in cross-striated muscle cells, whereas the two other filamin family members FLNa and FLNb are more ubiquitously expressed across tissues. Filamins generally function as structural scaffolds and signaling platforms, which is facilitated by their structure consisting of an amino-terminal actin-binding domain followed by 24 immunoglobulin-like (Ig-like) domains (d1-d24)^8^. Within this rod-like structure hinge regions provide additional flexibility, whereas the carboxy-terminal d24 enables filamin homodimerization for efficient actin cross-linking^9–11^. Based on its structural characteristics and its high dynamics, FLNc is essential for muscle development, Z-disc assembly, as well as the maintenance and repair of myofibrils which are continuously exposed to mechanical strain. Furthermore, loss of FLNc is embryonic lethal in mice^12^ and genetic mutations in *FLNC*are linked to skeletal myopathies and cardiomyopathies in humans^13–17^.

Specific molecular features such as the shuttling between Z-discs and the sarcolemma, the fast recruitment to sarcomeric lesions, or the binding of multiple proteins identify FLNc as a key signaling adaptor in myofibrils^15–17^. However, little is known about FLNc’s precise mode-of-action in mechano-signaling and its regulation under acute mechanical stress. A key region for mechano-sensing and many protein interactions is FLNc d20, which contains a unique 82 amino acid insert that is sufficient for Z-disc targeting^18^ and not found in FLNa or FLNb. During elevated and prolonged mechanical strain, unfolded and damaged FLNc is removed by chaperone-assisted selective autophagy (CASA), a process that is facilitated by binding of the CASA component BAG3 along with its partners HSP70 and HSPB8, and SYNPO2 (aka Myopodin) to FLNc’s mechanosensitive region^19–22^. Several other proteins are binding to this functionally important FLNc region such as HSPB1^23^, XIRP1^24^, NRAP^25^, and filamin-A-interacting protein 1 (FILIP1)^26^. However, how these multiple FLNc protein interactions are regulated, especially under conditions of acute mechanical stress, is largely unknown.

Several lines of evidence exist that phosphorylation provides a direct and tunable mechanism to reversibly regulate FLNc protein interactions, and thereby its functions, dynamics, and fate under the prevailing condition. First, phosphorylation of FLNc in the linker connecting Ig-like domains 23 and 24 by protein kinase C alpha (PKCα) was shown to protect it from calpain cleavage^27^. Second, phosphorylation promotes HSPB1 binding to FLNc’s mechanosensitive region to likely prevent over-extension under mechanical strain^23^. And third, protein kinase B (AKT)- and PKCα-mediated phosphorylation of mouse (m) FLNc-S2234/S2237 [orthologous sites in human (h) FLNc are S2233 and S2236] within its unique insert in d20 shields it from FILIP1-mediated degradation in mouse C2 skeletal myotubes^26^. Notably, phosphorylation of mFLNc at S2234 by AKT is highly efficient in mouse tissue^28^ and in cultured C2 myocytes, where it already occurs early during myogenesis as soon as FLNc levels increase^26^. Since FILIP1 binds FLNa and marks it for degradation in neural cells^29^, a picture has emerged in which FILIP1 specifically targets FLNa for degradation during myocyte differentiation, whereas FLNc is protected by d20 phosphorylation^26^.

Here, we investigated whether mechanical stress affects phosphorylation of mFLNc at S2234 in fully differentiated contracting C2 skeletal myotubes. To elicit acute mechanical stress, we subjected myotubes to electrical pulse stimulation (EPS) as an established in vitro system^30^. Quantitative phosphoprotein analysis demonstrated a drastic dephosphorylation of mFLNc-pS2234 and -pS2234/pS2237 despite high AKT activity in mechanically stressed myotubes. Protein interaction and biochemical studies revealed protein phosphatase 1 (PP1) to be responsible for mFLNc-pS2234 dephosphorylation. Additional binding studies confirmed a direct interaction between FLNc and FILIP1, which is higher for PP1c-catalyzed dephosphorylated mFLNc-S2234. As FILIP1 is a known factor for mediating filamin degradation^26,29^, our data suggest a counterplay between AKT and PP1 for determining the fate of FLNc under high mechanical stress.

## Results

### FLNc is dephosphorylated at S2234 in C2 myotubes during mechanical stress

**Fig. 1:**
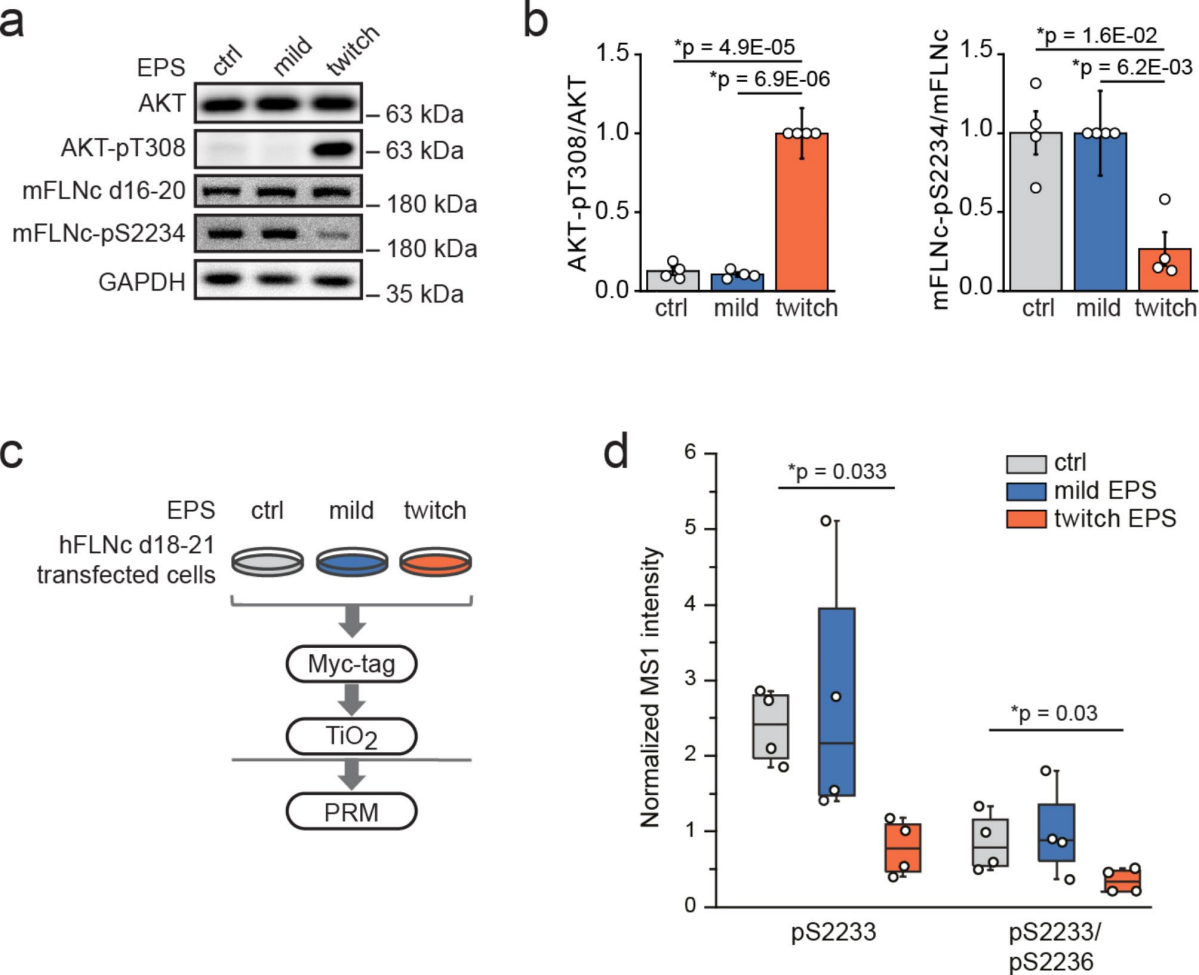
Mechanical stress leads to the dephosphorylation of mFLNc at pS2234. **(a)** Fully differentiated contracting C2 skeletal myotubes were subjected to mild EPS (3 h) or twitch EPS (3 h) for mechanical stress induction or non-treated as control. For Western blot analysis, myotube lysates were prepared and probed with total and phospho-specific antibodies as indicated. AKT-pT308 was used as readout for AKT activity. **(b)** Quantification of the Western blot data exemplarily shown in A. Calculated signal intensities were normalized to the control and a two-tailed paired student’s t-test was performed. Error bars represent the SEM (*n* = 4). **(c)** Experimental setup for the analysis of mFLNc d20 phosphorylation by mass spectrometry (MS). C2 cells were transfected with Myc-tagged hFLNc d18-24 (miniFLNc) and differentiated myotubes were subjected to mild or twitch EPS, or non-treated as control. For targeted MS analysis, hFLNc-d18-24 was enriched via Myc-tag and digested using trypsin followed by phosphopeptide enrichment using titanium dioxide (TiO_2_) beads and measured using parallel reaction monitoring (PRM). **(d) **PRM data displaying changes in phosphorylation of hFLNc at S2233 and S2233/S2236 determined by the measured MS1 intensities of the singly and doubly phosphorylated peptides LGpSFGSITR (p, phosphate moiety) and LGpSFGpSITR, respectively. PRM data were quantified using Skyline^101^ and normalized to an internal phosphopeptide standard. A two-tailed, paired student’s t-test was performed and data is presented as whisker plot (*n* = 4).

We previously showed that mouse/human FLNc is phosphorylated by AKT at S2234/S2233 in its mechanosensitive region, which protects it from FILIP1-mediated degradation in skeletal myocytes^26^. We hypothesized that mFLNc-S2234 phosphorylation is reversible to regulate FILIP1 binding under mechanical stress. To investigate this, we subjected C2 myotubes to EPS for 3 h adjusted to induce basal (mild EPS) or high intensity contractions (twitch EPS)^26,27,30^, or left them untreated as control. Through quantitative immunoblot analysis, we monitored AKT activity using T308 and S473 phosphorylation as readouts^31^. Whereas AKT activity was low in myotubes subjected to mild EPS or without EPS, it was strongly increased under twitch EPS (Fig. 1a and b, **Supplementary Fig. 1a and 1b**), in line with phospho-dependent AKT activation upon skeletal muscle contraction^32^. In accordance to published work^26^, mFLNc-S2234 phosphorylation was already detected at low AKT activity in C2 myotubes. Strikingly, despite high AKT activity under twitch EPS, mFLNc-S2234 phosphorylation was strongly decreased, without altered FLNc abundance (Fig. 1a and b). Of note, a phosphosite-specific antibody against mouse/human FLNc-S2237/2236 is not available.

To validate the unexpected finding of mFLNc-pS2234 dephosphorylation and also monitor the bis-phosphorylated form, we employed a targeted immunoprecipitation mass spectrometry (IP-MS) approach using parallel reaction monitoring (PRM) for phosphopeptide quantification (Fig. 1c). C2 myotubes were transfected with a myc-tagged hFLNc construct consisting of the actin-binding domain (ABD) and d18-24 (termed miniFLNc; **Supplementary Fig. 1c**) and subjected to mild or twitch EPS or left untreated. After anti-Myc IP of miniFLNc and TiO_2_-based phosphopeptide enrichment, PRM data confirmed a significant reduction of S2233 and S2233/S2236 phosphorylation based on the intensities measured for the mono-phosphorylated (pS2233) and bis-phosphorylated (pS2233/pS2236) hFLNc peptides (Fig. 1d, **Supplementary Table 1**). Since phosphorylation at S2236 was only detected in bis-phosphorylated hFLNc peptides, we concluded that both sites are dephosphorylated under mechanical stress. Our findings therefore suggest the dominant action of a counteracting protein phosphatase that specifically targets FLNc in its unique insert in d20 during mechanical stress.

### Phosphatase inhibition increases phosphorylation of mFLNc at S2234

**Fig. 2:**
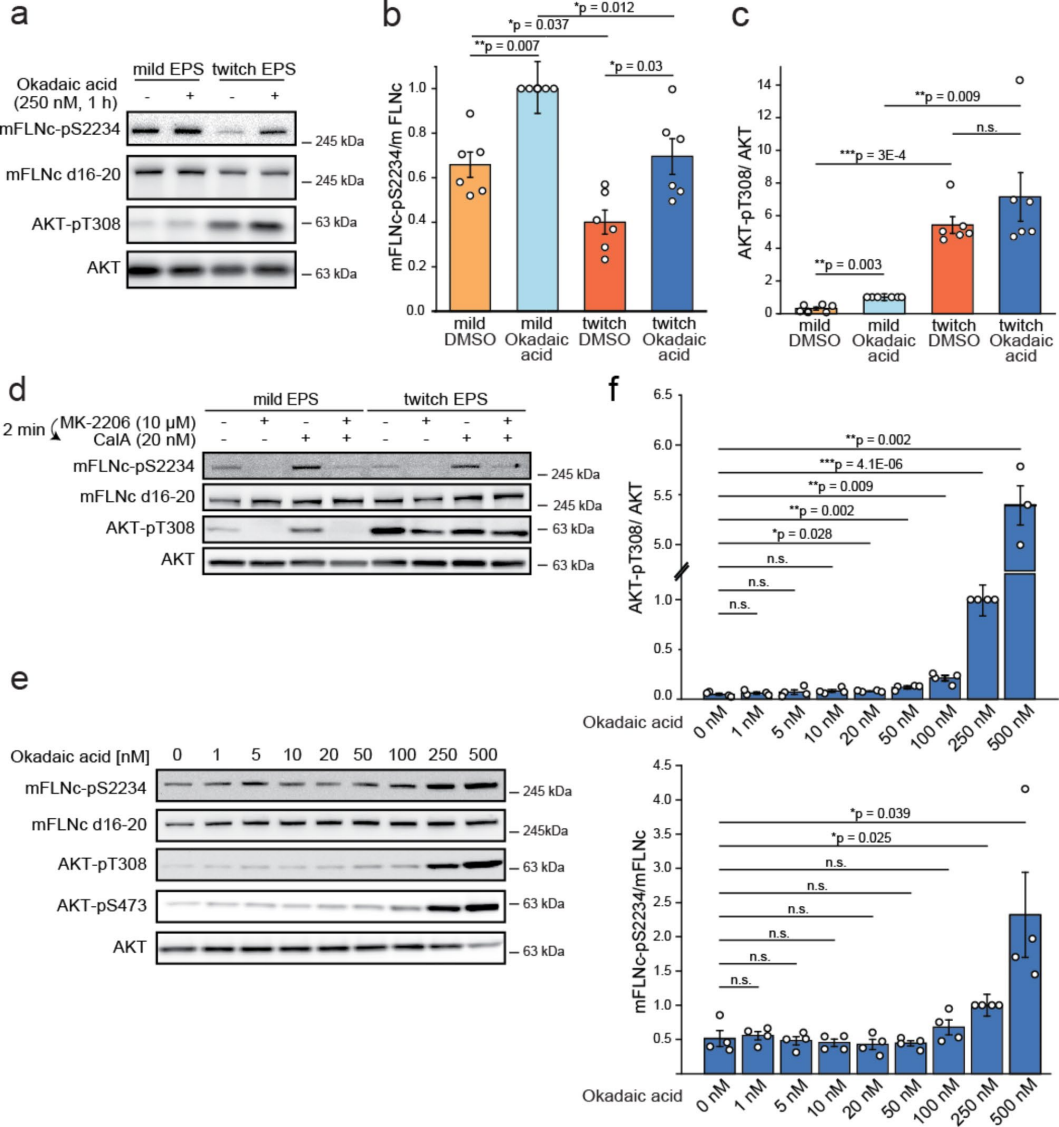
Regulation of mFLNc-S2234 phosphorylation through the counterplay of AKT and phosphoprotein phosphatase family members. **(a)** Mild or twitch EPS-treated or non-treated C2 myotubes were incubated with the phosphatase inhibitor okadaic acid as indicated. Cell lysates were analyzed by Western blotting using total and phospho-specific antibodies to display the activity of AKT and changes in the phosphorylation level of its substrate mFLNc-S2234. **(b**,** c)** Quantification of Western blot data shown in (a). Phospho-specific signal intensities were normalized to the respective total signal intensities. A two-tailed, paired student’s t-test was performed and error bars represent the SEM (*n* = 6). **(d)** Mild or twitch EPS-treated C2 myotubes were incubated either with the AKT inhibitor MK-2206 or the phosphatase inhibitor Calyculin A, or both modulators consecutively. The lysates were analyzed by Western blotting to monitor changes in the phosphorylation levels of AKT-pT308 and mFLNc-pS2234. The phosphorylation of mFLNc-pS2234 is regulated through an interplay of AKT and a phosphatase, which is inhibited by Calyculin A. **(e)** Mild EPS-treated C2 myotubes were treated with increasing concentrations of the phosphatase inhibitor okadaic acid as indicated. Cells were lysed and Western blot analysis was performed. Total and phospho-specific antibodies were used to monitor changes in the activity of AKT and in the phosphorylation of its substrate mFLNc-S2234. **(f)** Quantification of the Western blot data exemplarily shown in (e). Phospho-specific signal intensities of endogenous mFLNc-p2234 as well as AKT-pT308 and AKT-pS473 were normalized to the respective total signal intensities. A two-tailed, paired student’s t-test was performed and error bars represent the SEM (*n* = 4).

To examine whether mFLNc-pS2234 is dephosphorylated by an active protein phosphatase under mechanical stress, we subjected C2 myotubes to mild or twitch EPS while inhibiting phosphatase activity with the pan-inhibitor okadaic acid (OA)^33,34^. Quantitative immunoblot analysis revealed a significant increase in mFLNc-pS2234 levels for both mild and twitch EPS-treated myotubes when concurrently treated with OA (Fig. 2a and b). As expected, the AKT-pT308 signal was considerably increased in twitch versus mild EPS-treated myotubes (Fig. 2a and c). OA treatment resulted in increased AKT-T308 phosphorylation under mild EPS, but not twitch EPS, when compared to control cells (Fig. 2a and c). Similar results were obtained when mild and twitch EPS-treated C2 myotubes were exposed to calyculin A (CalA), another phosphatase inhibitor (**Supplementary Fig. 2a**)^33,34^.

AKT is a known target of Ser/Thr protein phosphatases to modulate its activity^35–37^. Thus, to show that the increase in mFLNc-pS2234 levels is caused by a phosphatase and not by increased AKT activity, we first inhibited AKT with its specific inhibitor MK-2206^38^, and then used CalA to inhibit phosphatase activity both in mild and twitch EPS-treated C2 myotubes. Independent of AKT activity, mFLNc-pS2234 levels were increased upon phosphatase inhibition (Fig. 2d). Since this increase appeared to result directly from phosphatase inhibition a competitive regulation of mFLNc-S2234 phosphorylation through AKT and a so far unknown phosphatase was implicated.

CalA and OA inhibit most of the phosphoprotein phosphatases (PPPs). However, whereas CalA exhibits comparable activity towards both PP1 and PP2A, OA inhibits PP2A more strongly than PP1 (IC_50_ PP2A: 0.5–1 nM; IC_50_PP1: 60–500 nM)^34,39^. Hence, PP2A substrates exhibit higher sensitivity towards lower concentrations of OA compared with substrates of other PPPs, such as PP1^34^. To determine whether mFLNc-pS2234 is a potential substrate of PP2A, we incubated mild EPS-treated C2 myotubes with different concentrations of OA and analyzed mFLNc-pS2234 levels by Western blot analysis. As a control, we monitored AKT-pT308 and AKT-pS473, with pT308 identified as exclusive PP2A substrate and pS473 as substrate of both PP2A and PP1^36,40–42^. For all three phosphorylation sites analyzed we found a signal increase in dependency of OA treatment (Fig. 2e). Quantification of Western blot data revealed that levels of the PP2A substrate sites AKT-pT308 and -pS473 significantly increased (1.6-fold) at 20 nM and 10 nM OA concentration, respectively (Fig. 2f and **Supplementary Fig. 2b)**. In contrast, mFLNc-S2234 phosphorylation was significantly increased (1.9-fold) at 250 nM OA concentration (Fig. 2f). We therefore concluded that mFLNc-pS2234 is not a substrate of PP2A (or PP2A-like phosphatase) but another PPP that is less sensitive to OA such as PP1^33^.

### PP1 dephosphorylates mFLNc at pS2234 in vitro

**Fig. 3:**
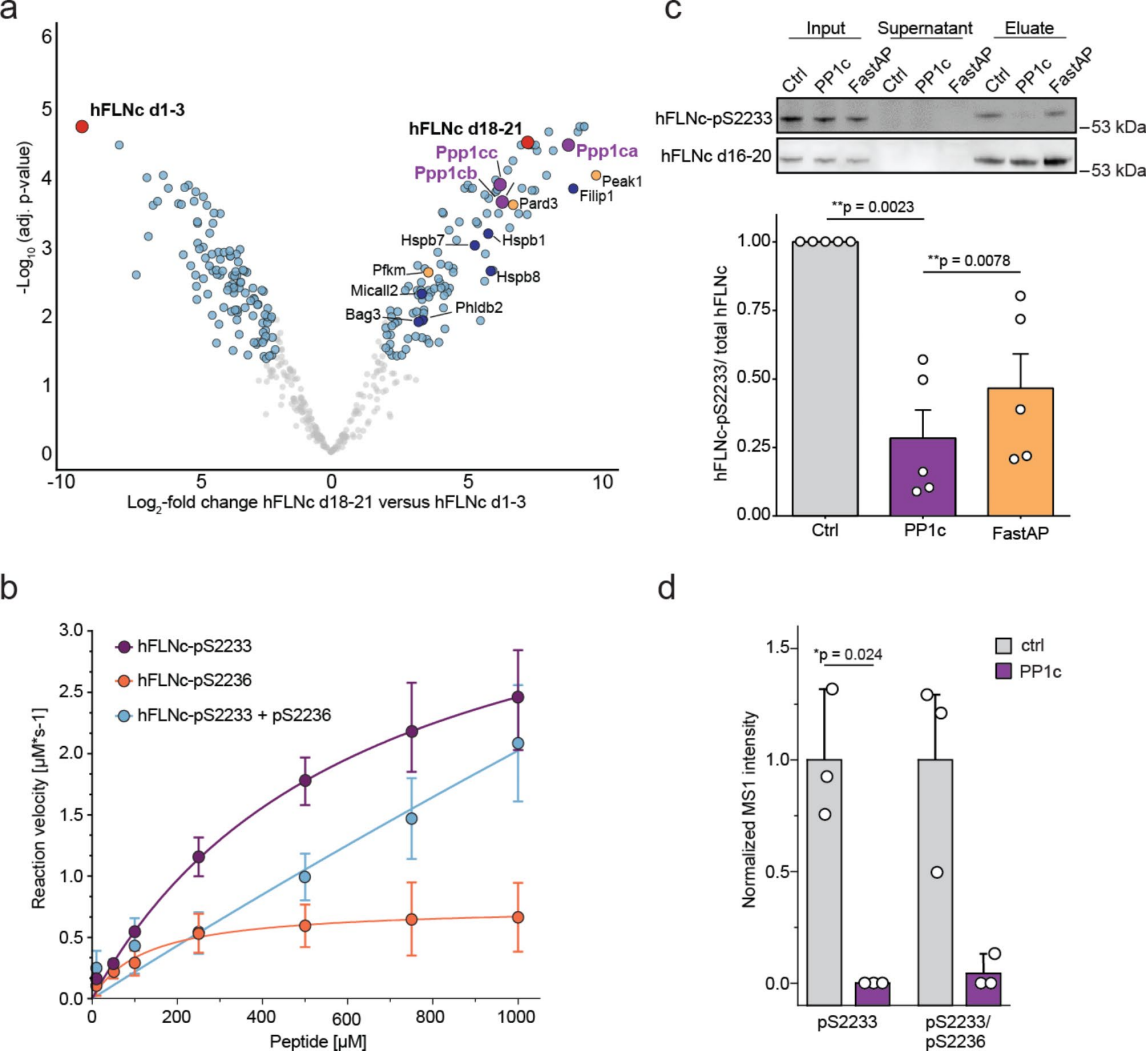
Identification of PP1 as a new putative hFLNc d18-21 binding partner and phosphatase of hFLNc-pS2233. **(a)** Recombinantly expressed His-tagged hFLNc d18–21 and His-tagged hFLNc d1–3 (control) bound to Ni^2+^-NTA agarose beads were incubated with C2 skeletal myotube lysate. Bound proteins were eluted and analyzed by LC-MS. Log_2_-fold changes of hFLNc d18-21 *versus* hFLNc d1-3 were plotted against -log_10_ adjusted p-values (*n* = 5). Proteins with a fold-change of + 4 or -4 and an adjusted p-value lower than 0.01 are shown in blue. Known FLNc interactors, dark blue; bait proteins in bold letters; PP1 catalytic subunits, red. Proteins are represented by their respective gene names. **(b)** Peptide-based phosphatase assay showing the dephosphorylation of 14-mer phosphopeptides of hFLNc amino acid sequence 2229–2242, either present as mono-phosphorylated (pS2233, pS2236) or bis-phosphorylated peptide versions. The rate of dephosphorylation by PP1 was monitored by detecting the phosphate release in a spectrophotometric assay. Error bars represent the SEM (*n* = 3). **(c)** FLAG-tagged hFLNc d18-21 was recombinantly expressed in HEK293 cells, immobilized with anti-FLAG beads and dephosphorylated on beads through PP1 or fast alkaline phosphatase (AP), which is used as an arbitrary phosphatase as control. Western blot analysis was performed to analyze hFLNc-S2233 phosphorylation levels. The phospho-specific signal of the control was set to 1, and the intensities were normalized to the respective total signal intensities relative to the control. A two-tailed, paired student’s t-test was carried out; error bars represent the SEM (*n* = 5). **(d)** Same assay as in (c) with PP1c-dependent dephosphorylation of hFLNc d18-21 followed by targeted phosphoproteomics analysis through parallel reaction monitoring (PRM). Protein digestion using trypsin was performed on beads followed by phosphopeptide enrichment using TiO_2_ beads before PRM analysis. Phosphopeptide intensities were normalized to the respective total signal intensities. A two-tailed, paired student’s t-test was performed (*n* = 3).

To obtain further insight whether PP1 is a phosphatase potentially responsible for the dephosphorylation of mFLNc-pS2234, we performed an exploratory pulldown experiment. To this end, recombinantly expressed His_6_-tagged hFLNc d18-21 or hFLNc d1-3 (control) were bound to Ni^2+^-NTA agarose beads and incubated with C2 myotube lysates followed by the identification of binding partners using quantitative MS analysis (Fig. 3a and **Supplementary Fig. 3a; Supplementary Table 2**). In this setting, the hFLNc domains are not phosphorylated due to the bacterial expression. However, PP1 can interact with its substrates in a phosphorylation-independent fashion, therefore not necessarily requiring a phosphorylation site for binding^43,44^. Among the significantly enriched proteins (fold-change ≥ 4, adjusted p-value < 0.01) were numerous known FLNc binding partners such as FILIP1^26^, HSPB1^23^or the CASA machinery constituents BAG3 and HSPB8^20^. In our search for potential phosphatases and regulatory proteins of phosphatases, we identified several regulatory interactors of protein phosphatase one (RIPPOs), specifically partitioning defective 3 homolog (PARD3), inactive tyrosine-protein kinase (PEAK1), and ATP-dependent 6-phosphofructokinase, muscle type (PFKM)^43^. Interestingly, all three catalytic subunits of PP1 (PP1c; PPP1CA, PPP1CB, PPP1CC) were significantly enriched with hFLNc d18-21, but not with hFLNc d1-3 (Fig. 3a). No other Ser/Thr phosphatase, or its regulatory proteins, was co-enriched with hFLNc d18-21 (**Supplementary Table 2**). Thus, the data suggests PP1c as a potential phosphatase directly targeting FLNc in its mechanosensitive region, which is in line with our quantitative Western blot data showing that mFLNc-pS2234 is sensitive to CalA but not OA (Fig. 2**)**.

To identify hFLNc-pS2233 as a potential substrate of PP1c, we designed a peptide-based phosphatase assay. For this, 14-mer phosphopeptides representing the hFLNc-pS2233, -pS2236, or -pS2233/pS2236 sites (sequences are identical for mFLNc) were synthesized (**Supplementary Fig. 3b**) and the enzymatic kinetics of recombinant PP1c (PPP1CA) towards these sites were determined through a spectrophotometric assay. Our data show that PP1c dephosphorylates all three hFLNc phosphopeptide variants at both phosphosites (pS2233, pS2236, pS2233/pS2236) in vitro, with the highest catalytic efficiency towards the hFLNc-pS2233 resembling phosphopeptide (Fig. 3b and **Supplementary Fig. 3b**). Interestingly, despite the reported low preference of PP1c towards negatively charged peptides^45^, PP1 also efficiently dephosphorylated the bis-phosphorylated peptide.

To further investigate PP1c-catalyzed FLNc dephosphorylation, FLAG-tagged hFLNc d18-21 was recombinantly expressed in HEK293T cells (**Supplementary Fig. 3c**), where it is also phosphorylated at S2233 and S2233/S2236^26^. After anti-FLAG IP, phosphorylated hFLNc d18-21 samples were incubated with recombinant PP1c or thermosensitive alkaline phosphatase (FastAP) as control (**Supplementary Fig. 3d**). Western blot analysis showed in vitro dephosphorylation of hFLNc-pS2233 through FastAP and PP1c in this simplified, non-competitive environment (Fig. 3c). Additional quantification of the immunoblot data revealed a significantly stronger dephosphorylation through PP1c compared to FastAP, which reinforces an efficient dephosphorylation mechanism as observed with the synthetic phosphopeptides (Fig. 3b). To confirm the immunoblot data and to additionally monitor the bis-phosphorylated hFLNc-pS2233/pS2236 form, we combined our phosphatase assay using PP1c and hFLNc-d18-21 with targeted MS analysis (**Supplementary Fig. 3d**). PRM data verified that PP1c efficiently dephosphorylates pS2233 and pS2233/2236 in the respective hFLNc phosphopeptides in vitro (Fig. 3d and **Supplementary Table 3**).

To conclude, our data show that PP1c is a so far unknown constituent of the hFLNc d18-21 interactome and efficiently dephosphorylates hFLNc at pS2233 and pS2233/pS2236 in vitro at the peptide and protein level.

### Activation of PP1 leads to the dephosphorylation of mFLNc at pS2234 in cells

**Fig. 4:**
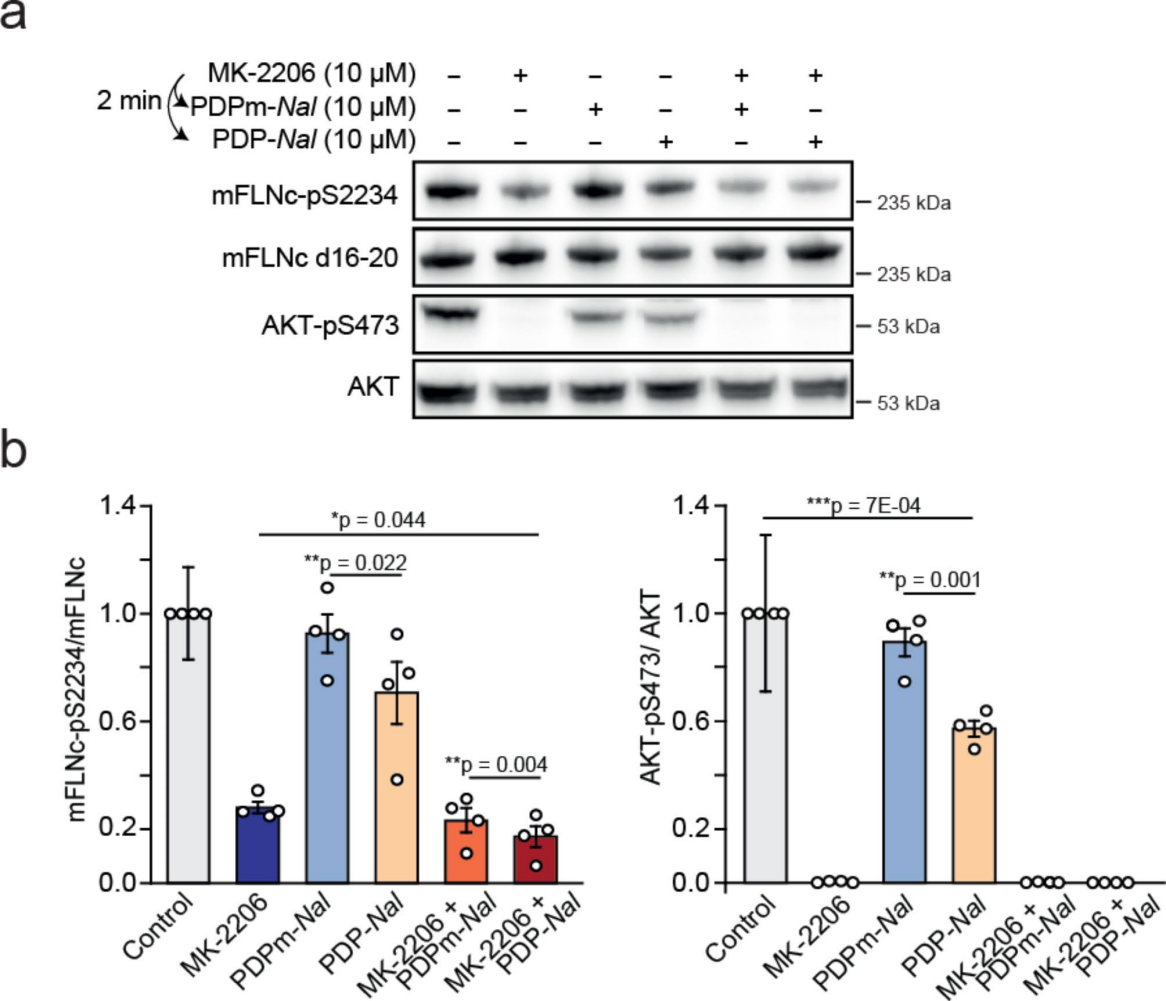
Activation of PP1 through PP1-disrupting peptides (PDPs) results in the dephosphorylation of mFLNc-pS2234 in C2 myotubes. **(a)** C2 myotubes were treated with combinations of the AKT inhibitor MK-2206, PP1-disrupting peptides PDP-*Nal*, and PDPm-*Nal* (control). Lysates were subjected to Western blot analysis using total and phospho-specific antibodies to display AKT-pS473 and mFLNc-pS2234 phosphorylation levels. **(b)** Quantification of immunoblot data exemplarily shown in (a). Phospho-specific signal intensities were normalized to the respective total signal intensities. A two-tailed, paired student’s t-test was carried out; error bars represent the SEM (*n* = 4).

To examine the role of PP1 for dephosphorylating endogenous full-length mFLNc in its mechanosensing d20 in a cellular system, we made use of PP1-disrupting peptides (PDPs)^46^. PDPs specifically release PP1 from its regulatory subunits to dephosphorylate substrates in close proximity^46,47^. In our approach, we used the optimized, photostable PDP containing 2-napthylalanine (PDP-*Nal*) and the inactive control peptide PDPm-*Nal*^48^. Our initial phosphatase inhibitor data (Fig. 2d**) **revealed that mFLNc-pS2234 is most likely regulated by the combined actions of a protein phosphatase and the basophilic kinase AKT. Consequently, the activation of PP1 alone would not be a conclusive approach, as it also leads to decreased activity of AKT through its dephosphorylation^36,49^. Accordingly, for C2 myotubes treated with PDP-*Nal,* but not PDPm-*Nal* (control), we observed a significant reduction in the phosphorylated form of both, the known PP1 substrate AKT-S473 and endogenous mFLNc-S2234 (Fig. 4a). To specifically measure the effect of PP1 on mFLNc-S2234 phosphorylation, we first inhibited AKT with MK-2206 and then added PDP-*Nal* for PP1 activation or PDPm-*Nal* as control. MK-2206 treatment abolished AKT activity as shown by non-phosphorylated S473. Quantitative analysis of the obtained Western blot data further revealed a significant decrease in mFLNc-pS2234 levels in C2 myotubes treated with MK-2206 and PDP-*Nal* compared to MK-2206 alone or with PDPm-*Nal* (Fig. 4a and b). These findings were consistently observed for FLAG-tagged hFLNc d18-21 (**Supplementary Fig. 3c**) immunopurified from HEK293 cells treated with MK-2206, followed by PDP-*Nal* or PDPm-*Nal* treatment (**Supplementary Fig. 4a and 4b**).

Taken together, our data corroborate a so far unknown role of PP1 in regulating FLNc phosphorylation in its mechanosensing d20 in contracting skeletal myocytes.

### PP1-catalyzed dephosphorylation of FLNc increases FILIP1 binding

**Fig. 5:**
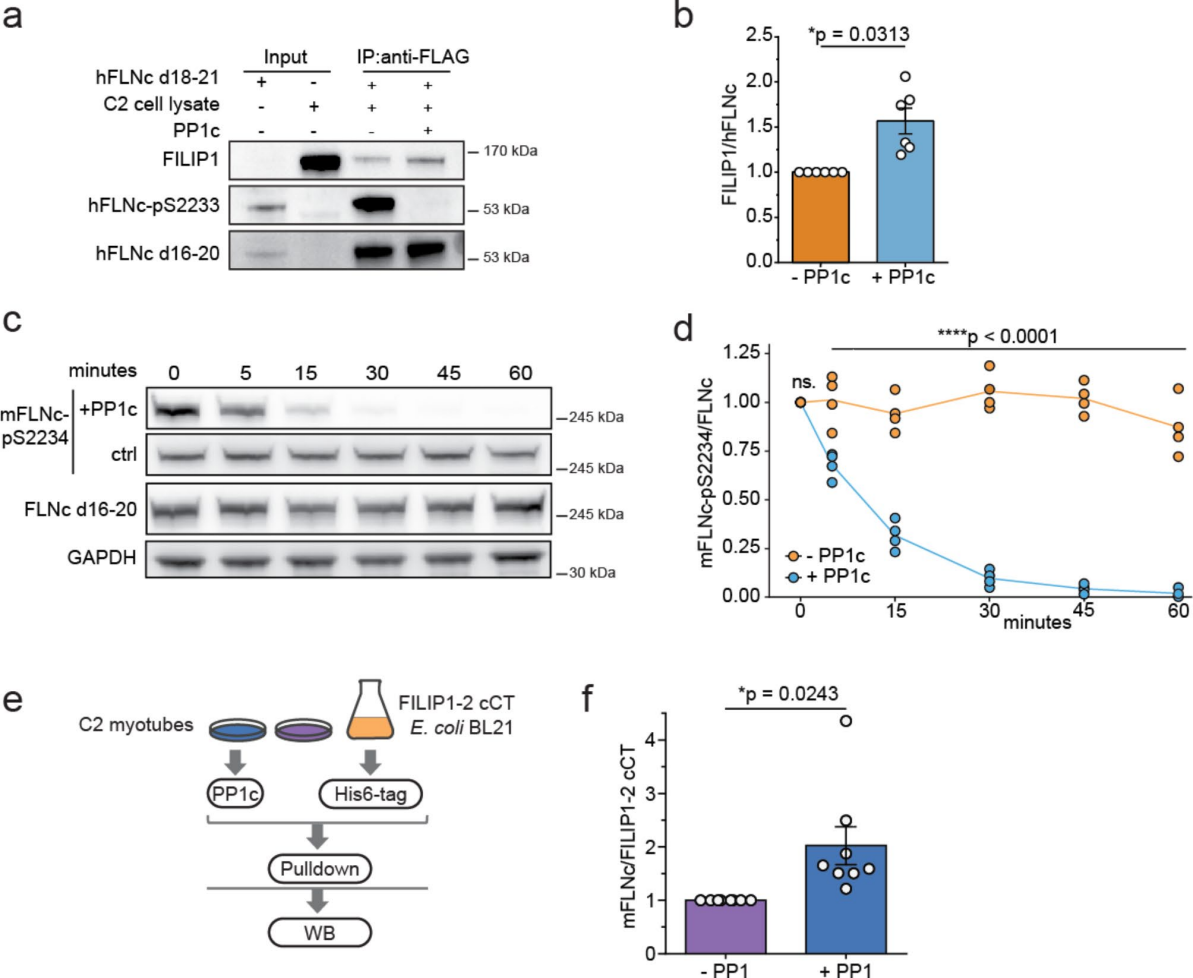
PP1-mediated dephosphorylation of human/mouse FLNc-S2233/2234 results in increased FILIP1 binding. **(a)** 3xFLAG-hFLNc d18-21 was expressed in HEK293 cells, single-step immunopurified via the FLAG tag, and treated on beads with or without recombinant PP1 followed by incubation with C2 myotube lysate. Western bot analysis of input samples and eluates was performed using specific antibodies against FILIP1, hFLNc d16-20 and hFLNc-pS2233 as indicated. **(b)** Quantification of the immunoblot data exemplarily shown in (a). FILIP1 signal intensities in eluates were normalized to total FLNc signals. SEM was calculated and a two-tailed student’s t-test was performed (*n* = 6). **(c)** C2 myotubes lysates were prepared and incubated with recombinant PP1c or without PP1c (control, ctrl) for different time points as indicated. Western blot analysis was performed to monitor the dephosphorylation of endogenous mFLNc-pS2234 with the respective phospho-site specific and total FLNc d16-20 antibody. **(d)** Quantification of immunoblot data shown in (c). Intensities of the mFLNc-pS2234 signals were normalized to the total mFLNc signal intensities of the respective treatment. Replicates are depicted as circles (*n* = 4), with the mean represented by a connecting line. A two-way ANOVA was carried out to determine significant changes in mFLNc-S2234 phosphorylation levels upon PP1c addition. **(e)** Experimental workflow to study the binding of endogenous mFLNc from C2 myotubes to the C-terminus of FILIP1 in dependency of PP1-mediated changes in mFLNc-S2234 phosphorylation levels. The carboxy-terminus of FILIP1-2-cCT-6xHis was recombinantly expressed in *E. coli* cells, bound to Ni^2+^-NTA agarose beads, and incubated with C2 skeletal myotube lysate that was incubated without or with recombinant PP1c for dephosphorylation of endogenous mFLNc-pS2234. The amount of FLNc recovered with the carboxy-terminus of FILIP1-2 cCT after pulldown was analyzed by Western blotting **(f)** PP1c-dependent changes in the amount of FLNc recovered with FILIP1-2 cCT following the experimental workflow shown in (e). Quantification is based on Western blot data shown in Supplementary Fig. 5c. FLNc signals were normalized to the levels of pre-coupled FILIP1-2 cCT. A two-tailed student’s t-test was performed, and error bars represent the SEM (*n* = 8).

We previously identified FILIP1 as a phosphorylation-dependent binding partner of FLNc which is involved in its degradation^26^. However, how the FLNc-FILIP1 interaction is reversibly regulated remained unknown. To address this gap in knowledge, we analyzed the binding of endogenous FILIP1 to hFLNc d18-21 in dependency of PP1c. Pulldown experiments were performed with immunopurified FLAG-tagged hFLNc d18-21 (**Supplementary Fig. 3c**), treated with or without recombinant PP1c and subsequently incubated with FILIP1-containing C2 myotube lysate (Fig. 5a, **Supplementary Fig. 5a**). Differences in the extent of binding of FILIP1 to phosphorylated or PP1c-dephosphorylated hFLNc d18-21 were determined through Western blot analysis. The PP1c treatment efficiently dephosphorylated hFLNc-pS2233 and resulted in an increase in FILIP1 binding compared to the untreated control sample (Fig. 5a). Quantitative data analysis confirmed the increase in the binding of endogenous FILIP1 to S2233 dephosphorylated hFLNc d18-21 (Fig. 5b).

To confirm the phosphoregulated binding of FILIP1 to FLNc, we examined the dephosphorylation dynamics of endogenous mFLNc-pS2234 in C2 myotubes. To this end, a time-course experiment was carried out by incubating C2 myotube lysate with recombinant PP1c between 0 and 60 min and monitoring pS2234 dephosphorylation levels of full-length FLNc over time (Fig. 5c). As a control, myotube lysates were treated with 20 nM CalA to prevent the dephosphorylation of mFLNc-pS2234 by endogenous phosphatases (Fig. 5c). In the PP1c-treated samples, mFLNc-pS2234 was significantly dephosphorylated after 5 min and after 60 min the reaction was complete (Fig. 5d). Next, we analyzed the binding of endogenous full-length mFLNc to FILIP1 in dependency of PP1c. To this end, we expressed a His6-tagged C-terminal FILIP1 construct (FILIP1-2 cCT)^26^, which contains the binding region for FLNc, and pre-coupled it to Ni-NTA beads for pulldown experiments with C2 myotube lysate pretreated with or without PP1c (Fig. 5e). Complete mFLNc-pS2234 dephosphorylation was confirmed in PP1c-treated myotube lysates (**Supplementary Fig. 5b**). Importantly, the binding of endogenous mFLNc to FILIP1-2 cCT increased significantly upon PP1c-catalyzed dephosphorylation compared to non-treated control lysate as shown by quantitative Western blot analysis (Fig. 5f and **Supplementary Fig. 5c**).

Collectively, our data show that PP1 efficiently dephosphorylates mFLNc-pS2234 in C2 myotubes and thereby provides a mechanism to regulate the binding of FILIP1 to FLNc under mechanical stress conditions.

## Discussion

**Fig. 6:**
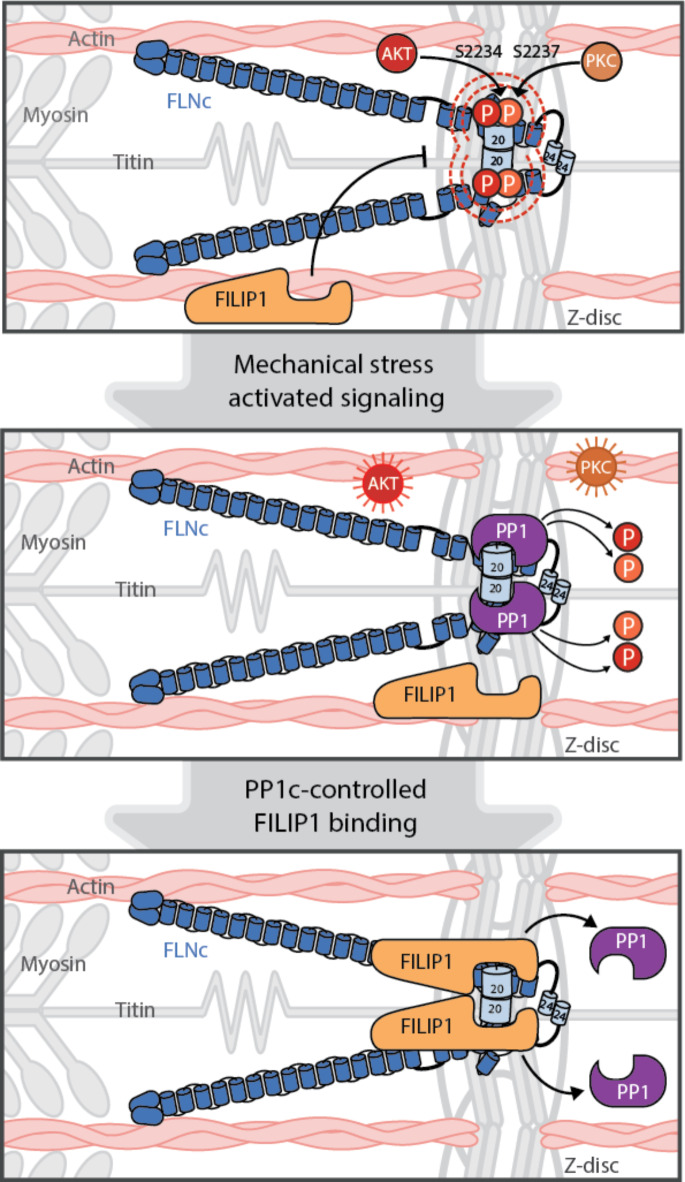
Illustrative summary of the regulation of mFLNc-S2234 and -pS2237 phosphorylation controlled by AKT and PKCα and the dominant action of PP1c in skeletal myotubes under non-stressed and acute mechanical stress conditions. **Top**, Under non-stressed conditions, AKT and PKC**α**-mediated phosphorylation of mFLNc at S2234 and S2237 impedes FILIP1 binding to its mechanosensitive region. **Middle**, During acute mechanical stress, mFLNc is dephosphorylated at pS2234/pS2237 through the dominant action of the protein phosphatase PP1 despite increased AKT and PKC**α** activity. **Bottom**, PP1-controlled dephosporylation of FLNc results in increased binding of FILIP1, a factor mediating FLNc degradation^26^.

Preceding work has shown that FLNc is a multi-phosphorylated protein, which is under the control of different protein kinases to modulate its protein interactions, dynamics, and degradation in cross-striated muscle cells^26,27,50^. Here, we report for the first time that FLNc is a substrate of the protein phosphatase PP1c in mechanically stressed cultured skeletal myocytes. Based on the findings in this study and previous data^26^, we propose a reversible mechanism how FLNc phosphorylation in its mechanosensitive domain is regulated to promote FILIP1 binding in mechanically stressed C2 myotubes (Fig. 6). Under conditions of basal contractions, mFLNc is phosphorylated within its unique insert in d20 at S2234 by AKT^26^. Together with PKCα-mediated phosphorylation of S2237, S2234 phosphorylation diminishes the binding of FILIP1 to mFLNc under basal conditions (Fig. 6, top). In contrast, under acute mechanical stress, PP1 is active and efficiently dephosphorylates mFLNc at pS2234 and pS2237 despite high kinase activity (Fig. 6, middle). This PP1-catalyzed site-specific dephosphorylation of FLNc facilitates increased FILIP1 binding (Fig. 6, bottom), a factor mediating FLNc degradation^26^.

We show that basal contractions induced in vitro by mild EPS only elicits low levels of AKT activity, whereas twitch EPS leads to a strong increase in AKT activity in C2 skeletal myotubes **(**Fig. 1a and b). These findings are in line with previous reports on low AKT activity in non-exercised human muscle^51^, non-differentiated myoblasts^28^ and differentiated skeletal myotubes without electrical stimulation^52,53^. In contrast, muscle contraction strongly provokes AKT activity^32^. Furthermore, our data confirm that low AKT activity is sufficient to efficiently phosphorylate mFLNc-S2234 as also observed in skeletal muscle tissue^28^, differentiating C2 skeletal myocytes, and fully differentiated contracting myotubes under basal condition^26^. Our finding that mFLNc-S2234 phosphorylation is strongly decreased under mechanical stress, despite high AKT activity, was unexpected and suggested the dominant action of a mechanical stress-activated protein phosphatase **(**Fig. 1a and b). In line with this assumption, mFLNc-pS2234 levels were increased in mild and twitch EPS-treated C2 myotubes when applying the PPP inhibitors OA at high concentration and CalA at lower concentration with or without preceding AKT inhibition (Fig. 2a and d). The latter finding supports that FLNc d20 is a direct PPP target. Although small molecule phosphatase inhibitors such as OA and CalA lack specificity towards a single PPP^34,44^, our titration experiments enabled us to narrow down the set of candidate PPPs when comparing the sensitivity of different substrate sites to these inhibitors. Based on the weak sensitivity of mFLNc-pS2234 towards OA, compared to the known PP2A substrate AKT-pT308^42^ (Fig. 2e and f, **Supplementary Fig. 2b**), PP2A-like PPPs (PP2A, PP4, PP6)^34^ could be excluded for targeting the pS2234 site.

AKT is known to be inactivated by PP1^37^. Therefore, our finding of high AKT activity together with PP1 activity on mFLNc-pS2234 may at first sight appear contradictive. However, more than 200 PP1-holoenzymes regulate PP1’s activity and specificity, and PP1-holoenzymes can also be modulated by other specific regulatory factors including inhibitory proteins or posttranslational modifications^44,54–57^. Accordingly, it is evident that when PP1 is activated toward a certain substrate, here FLNc-pS2234, it is not automatically activated toward a different substrate, such as AKT.

The phosphorylation site S2234/S2233 in FLNc’s unique insert in d20 is conserved in all mammalian species. This molecular region is sufficient to direct FLNc to the Z-disc^58^ and is involved in a number of protein interactions (e.g., Bag3^35^, Hspb1^23^, FILIP1^26^, myotilin). Moreover, our data underscore the role of FLNc as a key signaling adaptor in skeletal muscle cells, since in addition to being a target of several kinases (e.g. PKCα^27^, AKT^28^) or a scaffold protein for MEK1/2 and ERK1/2 activation^59^, it also interacts with PP1 subunits (Fig. 3a). In line with our phosphatase inhibitor findings (Fig. 2), our data suggest a FLNc d18-21 domain-specific binding of all three catalytic subunits of PP1 (Ppp1ca, Ppp1cb, Ppp1cc). The mouse and human FLNc-pS2234/pS2233 site is part of a basophilic RxRxxp[S/T] motif^26,60^, which is assigned to the class of AKT kinases and comprises the shorter arginine-directed motif Rxxp[S/T] motif targeted by PP1^45,61,62^. An arginine residue in close proximity (favored in position -3) to the target phosphosite directly binds to the acidic groove of PP1c that is located adjacent to its active site^61,63^. Accordingly, the 14-mer hFLNc-pS2233 phosphopeptide with an arginine in the -3 position showed the fastest PP1c-mediated dephosphorylation dynamics (Fig. 3b). Our in vitro assay further revealed that recombinant PP1c is also capable to dephosphorylate the pS2233/pS2236 bis-phosphorylated hFLNc peptide with the latter site being a known substrate of PKCα^26^ (Fig. 3b and d, **Supplementary Fig. 3b**). This finding is supported by our quantitative PRM data demonstrating hFLNc-pS2233/pS2236 dephosphorylation during twitch EPS **(**Fig. 1d),

In a cellular context, PP1 is typically regulated by RIPPOs, which includes RIPPO-driven PP1 targeting and substrate interaction^43,44^. In our hFLNc d18-21 interactome, we found the known RIPPOs PARD3, PEAK1, PFKM^43^. However, the high enrichment of all three PP1cs suggests the possibility that binding of PP1c to FLNc might not necessarily require a RIPPO (Fig. 3a). Hence, it will be of interest to explore the existence of a potential RIPPO-independent mode of action for PP1 in future work.

PDPs were shown to disrupt PP1 holoenzyme formation with nanomolar potency in vitro, thereby liberating PP1 that dephosphorylates nearby substrates in cells when using the optimized PDP-*Nal*version^46,48,64^. Treatment of C2 myotubes or HEK293 cells with PDP-*Nal*, but not the control peptide PDPm-*Nal*, resulted in decreased mFLNc-pS2234 and hFLNc-pS2233 levels, respectively (Fig. 4, **Supplementary Fig. 4**). Since phospho-signaling can be rapid^65^, this decrease in mouse/human FLNc-pS2234/pS2233 levels may also be the result of an indirect effect of PP1 by dephosphorylating AKT-pS473, which inactivates AKT. However, inhibiting AKT by a drug prior to PDP treatment demonstrated that PP1 exerts a direct effect on FLNc and specifically dephosphorylates mFLNc-pS2234 in C2 myotubes and hFLNC-pS2233 in HEK293 cells (Fig. 4, **Supplementary Fig. 4**). Finally, our data revealed an increased FLNc-FILIP1 interaction upon PP1c-mediated dephosphorylation of both endogenous full-length mFLNc and hFLNc d18-21 (Fig. 5). These findings are in line with previous work showing increased FILIP1 binding to phospho-dead FLNc mutants, resulting in FILIP1-mediated FLNc degradation in C2 skeletal myotubes^26^.

What is the functional relevance of such a reversible phospho-regulation of FLNc within its unique insert in d20? First, mFLNc-S2234 is a high-quality substrate of AKT^26^, and thus phosphorylated even at low AKT activity in non-stressed skeletal myotubes. Second, high AKT activity elicited by contractions in skeletal muscle^32^ is beneficial, as the AKT-mTORC1 signaling axis drives protein synthesis and muscle growth^52,66,67^. Thus, while highly active AKT is needed for maintaining skeletal myotube functions, the dominant and specific action of PP1c enables to precisely target and dephosphorylate mFLNc-pS2234 during mechanical stress. Notably, FLNa and FLNb are not affected as they lack the 82 amino acid stretch harboring S2234 (and S2237) in mFLNc. Third, this PP1c-dependent mechanism elevates FILIP1 binding to the mechanosensitive region of FLNc under acute mechanical stress. However, a limitation of this study is that it is based on an in vitro model using cultured skeletal myocytes subjected to EPS to induce mechanical stress. To substantiate the physiological relevance of this phospho-regulated mechanism of FLNc-FILIP1 binding, we envision the generation of human pluripotent stem-cell-derived skeletal myocytes endogenously expressing the respective FLNc-phospho-variants and a matching *FLNC* mouse model. Interestingly, a *FLNC*W2164C mouse model, which carries the missense mutation also in d20, has been associated with hypertrophic cardiomyopathy, and shows increased expression, higher dynamics, and altered localization of the mutant protein^68^. In C2 myotubes, we found that S2234/S2236 phosphorylation stabilizes FLNc and ensures its high dynamics and mobility in contracting, non-stressed skeletal myotubes^26^. Thus, site-specific changes in FLNc’s mechanosensitive d20 through point mutations or (de)phosphorylation can directly affect its functions and dynamics.

Previous^13,69,70^ work showed that mechanical force in the physiological range is capable of unfolding the filamin domain pair d20-d21^71,72^ and subsequently unfolded FLNc is a client of the CASA machinery^19–21^. The latter is important to prevent the aggregation of partially unfolded FLNc, which may occur in the context of myofibrillar lesions that are elicited upon eccentric exercise^30^. The high physiological significance of this phenomenon is highlighted by a number of myofibrillar myopathies (MFMs) in patients, in which *FLNC *point mutations potentiate FLNc aggregation, thereby causing an immense formation of larger protein aggregates and impaired protein degradation, which in turn aggravates lesion formation and ultimately leads to the disintegration of myofibrils, muscle weakness, and premature death^73–75^. The finding that misfolded, dysfunctional FLNc leads to the formation of sarcomeric lesions, demonstrates its importance for myofibrillar Z-disc stabilization and maintenance^75,76^. Notably, this Z-disc instability caused by FLNC mutations is intensified by acute exercise in mouse and filaminopathy patients^75^.

Despite its large size, functional FLNc is highly dynamic and mobile, and has been shown to immediately relocate to sarcomeric lesions in striated muscle cells^77^. Thus, FLNc and its binding partners (XIRP proteins, aciculin) are deemed as specific markers for myofibrillar lesions^30,78–80^. Like FLNc, FILIP1 is increasingly expressed during myogenic differentiation, and localizes to Z-discs during maturation of myofibrils^26,81^. FILIP also rapidly translocates to sarcomeric lesions, where it concentrates over time^81^. Thus, the FLNc-FILIP1 axis is not only vital for Z-disc maturation and stabilization but also for repairing damaged sarcomeres. FILIP’s functional importance has recently been recognized in patients carrying homozygous FILIP1 variants, who show various neurological symptoms, including MFM and dysmorphic features^82^. The identified pathogenic variants of FILIP1 were accompanied with FLNc dysregulation, and manifested in myofibrillar disintegration, altered Z-disc architecture and protein aggregates known for MFMs. Consistently, aggregated FLNc co-localized with FILIP1 missense variant in damaged patient muscle tissue, which showed that FLNc degradation was impaired^82^. These findings further highlight the important role of the FLNc-FILIP1 axis for healthy muscle structure and function in humans. Based on the biochemical studies presented in this work using an in vitro model, we conclude that PP1c-controlled binding of FILIP1 to FLNc’s mechanosensitive region provides a regulatable direct mechanism for recognizing unfolded, damaged FLNc for fast removal in skeletal myotubes under acute mechanical stress.

## Methods

### Cell culture

C2 myoblasts(gift from D. Fürst; Institute of Cell Biology, University of Bonn, Germany) have the accession number CVCL_6812 in the Cellosaurus database (www.cellosaurus.org) and were first described by Yaffe and Saxel^83^. C2 myoblasts with a passage number not exceeding 20 passages were used in experiments and checked for cell growth and morphology. C2 cells were cultured in high-glucose Dulbecco’s modified Eagle medium (DMEM) GlutaMAX™ (Gibco/Thermo Fisher Scientific, Darmstadt, Germany) supplemented with 15% (v/v) fetal bovine serum (FBS; Sigma-Aldrich/Merck, Darmstadt, Germany), 2% (v/v) sodium pyruvate, and 1% (v/v) non-essential amino acids (both Gibco/Thermo Fisher Scientific). Myoblasts were seeded into 6-well plates (Corning Incorporated, New York, USA), grown to confluency, and differentiated into myotubes by reducing the serum content to 2% horse serum (Sigma-Aldrich/Merck) and omitting sodium pyruvate from the medium. Differentiation medium was changed every 48 h until cells were fully differentiated after 3–4 days. The differentiation status and the formation of contracting C2 myotubes was monitored by light microscopy. Transient transfections of C2 cells were performed as described before^26^.

Human embryonic kidney 293T (HEK293T; obtained from EMBL cell line repository) cells were cultured in high-glucose DMEM supplemented with 10% (v/v) FBS and 1% (v/v) penicillin/streptomycin (Gibco/Thermo Fisher Scientific). Transient transfection (for 24 h) of HEK293T cells was carried out at 40–50% confluency using FuGene HD transfection reagent (Promega, Mannheim, Germany) and followed the manufacturer’s instructions using OptiMEM (Gibco/Thermo Fisher Scientific) and a plasmid-to-transfection reagent ratio of 1:6 (w/v).

All cells were regularly tested for contamination with mycoplasma and were found to be mycoplasma-free.

For in cell kinase or phosphatase stimulation, the following inhibitors were used: MK-2206 (10 µM, Selleckchem, Houston, USA), Calyculin A (20 nM; Cell Signaling Technology), or okadaic acid (250 nM; Merck KGaA, Darmstadt, Germany). For phosphatase activation, PDP-*Nal* or the control PDPm-*Nal*was used (10 µM; synthesized as described^48^).

## Electrical pulse stimulation of C2 skeletal muscle cells

C2 myotubes were serum-starved in DMEM GlutaMAX™ containing 1% non-essential amino acids for 4 h before EPS was applied as mild stimulus (i.e., mild EPS; 0.05 Hz, 4 ms, 10 V) or twitch stimulus (i.e., twitch EPS; 1 Hz, 20 ms, 10 V) for 3 h using a C-Pace EP Culture Pacer (IonOptix, Milton, USA). Serum-starved, non-stimulated myotubes served as control. For phosphatase inhibitor experiments using okadaic acid, cells were serum-starved for 3 h and cells were pre-stimulated with mild EPS for 1 h prior to mild/twitch stimulation for 3 h.

## Cell lysis

Unless described otherwise, C2 cells were washed twice with cold PBS and lysed by sonication (30 s) on ice using RIPA buffer (50 mM Tris, 150 mM NaCl, 0.1% [w/v] sodium dodecyl sulfate [SDS], 0.5% [w/v] sodium deoxycholate, 1% [v/v] Triton X-100, pH 7.5) supplemented with protease and phosphatase inhibitors (protease inhibitor cocktail [Roche Diagnostics, Mannheim, Germany], 1 mM sodium orthovanadate, 10 mM β-glycerophosphate, 9.5 mM sodium fluoride, 10 mM sodium pyrophosphate). Insoluble material was removed by centrifugation (21,000 x g, 20 min, 4 °C).

HEK293T cells were lysed in HEK293T lysis buffer (100 mM NaCl, 10 mM Tris, 0.1% [v/v] IGEPAL, 1 mM EDTA, 1 mM EGTA, 20 nM Calyculin A, 1x cOmplete Mini protease inhibitor cocktail [Sigma-Aldrich], 1 mM DTT, pH 7.4). Cells were scraped from plates, lysed by 10 pushes through an injection needle (21 G, BD Braun), and insoluble material was pelleted by centrifugation (10 min, 10,000 x g, 4 °C).

The protein concentration of cleared cell lysates was determined using the BCA assay (Thermo Fisher Scientific) according to the manufacturer’s instructions.

### Cloning of plasmids

FLNc d18-21-His-EEF, cloned into the pcDNA3.1 vector, FLNc d1-3-His, cloned into the pET23a vector, and His-FILIP1-2 cCT, cloned into the pET23aT7 vector for bacterial expression were obtained from the lab of Prof. Dr. Dieter Fürst (Department of Molecular Cell Biology, University of Bonn, Germany). The origin of the human FLNc complementary DNA (cDNA) and cloning procedures were described previously^26,27^. Further FLNc constructs were cloned based on these DNA sequences. Myc-miniFLNc d18-24-V5, containing a Myc tag, was cloned into a pcDNA3.1 vector for mammalian cell transduction by Gibson assembly^84^, using appropriate primers. The sequence of FLNc d18-21-His-EEF was inserted into the pCMV-3Tag-A1 vector resulting in a final construct of 3xFLAG-FLNc d18-21-His-EEF, which was used for transient transfection of HEK293T cells.

## Gel electrophoresis and immunoblotting

Proteins were separated by SDS polyacrylamide gel electrophoresis (SDS-PAGE) using 4–12% NuPAGE™ Bis-Tris gradient gels (Life Technologies/Thermo Fisher Scientific) according to the manufacturer’s instructions or 8% polyacrylamide gels. Proteins were visualized using colloidal Coomassie Brilliant Blue or transferred onto PVDF membranes for subsequent immunoblotting. The following primary antibodies were used: anti-Akt (1:1,000, #4691), anti-Akt-pT308 (1:1,000, #2965), anti-Akt-pS473 (1:1,000, #4060), and anti-GAPDH (1:1,000, #2118) from Cell Signaling Technology (Leiden, The Netherlands), anti-FLNc-pS2233 (0.2 µg/mL) from Kinasource Limited (Dundee, UK), and anti-FLNc d16-20 (1:50,000)^74^. Horseradish peroxidase-conjugated secondary anti-rabbit (1:10,000, #A0545) and anti-sheep (1:10,000, #713-035-147) antibodies were purchased from Sigma-Aldrich/Merck and Dianova (Hamburg, Germany), respectively.

Immunoblot signals were quantified using Quantity One (BioRad, version 4.6.8) or Fiji/ImageJ (version 2.0.0-rc-34/1.50a)^85^ and GraphPad Prism v6 (GraphPad Software, Inc.). Background signals were subtracted, and phospho-to-total protein signals were calculated. Ratios were then normalized to the control sample and two-sided, paired Student’s t-tests were performed to determine statistical significance.

## Immunoprecipitation of myc-tagged miniFLNcd18-24

Cleared lysates of mild or twitch EPS-treated or untreated differentiated C2 cells transiently transfected with Myc-miniFLNcd18-24 (one 6-well plate per replicate) were mixed with 40 µL of Myc-Dynabeads (Invitrogen/Thermo Fisher Scientific), and Myc-miniFLNcd18-24 was enriched according to the manufacturer´s instructions. Samples were analyzed by PRM. To this end, proteins were on-bead reduced, alkylated and digested using trypsin (1 µg sequencing grade trypsin [Promega] per sample), followed by TiO_2_-based enrichment of phosphopeptides employing the EasyPhos method as described before^86^.

### FLNc d18-21 and FLNc d1-3 pulldown experiments

For pulldown experiments of FLNc d18-21 and FLNc d1-3 (the latter serving as control), FLNc d18-21-His-EEF and FLNc d1-3-His were recombinantly expressed in *E.coli* BL21(DE3) and immobilized on Ni^2+^-NTA agarose beads (Qiagen, Hilden, Germany) as described previously^26^. Differentiated C2 cells were lysed by sonication (10 s) using co-immunoprecipitation (co-IP) buffer (25 mM Tris, 150 mM NaCl, 0.5% [w/v] sodium deoxycholate, 1% [v/v] Nonidet P-40, 10% [v/v] glycerol, 2 mM EDTA, protease inhibitor cocktail tablet [Roche Diagnostics], pH 8.0). Insoluble material was removed by centrifugation (21,000 x g, 10 min, 4 °C). Immobilized FLNc d18-21 and FLNc d1-3 were washed twice with co-IP buffer, mixed with cleared C2 cell lysates, and incubated for 1 h under constant agitation at 10 °C. Beads were collected by centrifugation (700 x g, 2 min, 4 °C) and washed 5 times with co-IP buffer. To elute proteins bound to the beads, SDS sample buffer (2% [w/v] SDS, 5% [v/v] β-mercaptoethanol, 10% [v/v] glycerol, 250 mM Tris/HCl pH 6.8, bromphenol blue) was added and samples were boiled for 5 min at 95 °C. Proteins were separated by SDS-PAGE and visualized using colloidal Coomassie Brilliant Blue. Gel lanes were cut into eight slices each and processed for LC-MS analysis of the proteins essentially as described before^87^ including reduction of cysteine residues, alkylation of free thiol groups, and tryptic in-gel digestion. Peptide mixtures were desalted using C18 StageTips^88^, dried *in vacuo* and reconstituted in 0.1% trifluoroacetic acid (TFA) for LC-MS analysis.

### FLNc d18-21 dephosphorylation and pulldown experiments

For the dephosphorylation of FLAG-tagged FLNc d18-21 expressed in HEK293T cells, cleared lysates of cells (2 mg of protein per sample) transiently transfected with the 3xFLAG-FLNc d18-21-His-EEF vector were first mixed with 25 µL of Anti-FLAG^®^ M2 Magnetic Beads (Sigma Aldrich) and incubated end-over-end for 2 h at 4 °C. Unbound material was discarded and beads were washed twice each with HEK293T lysis buffer and phosphatase reaction buffer (20 mM Tris pH 7, 100 mM NaCl, 1 mM DTT, 1 mM MnCl_2_). FLNc d18-21 was then dephosphorylated on beads in a total volume of 50 µL phosphatase reaction buffer containing 1 µM PP1CA or 5 U FastAP Thermosensitive Alkaline Phosphatase (Thermo Fisher Scientific) or left untreated (control). Samples were incubated for 30 min at 30 °C and 200 rpm. The supernatant was discarded. Beads were washed twice with phosphatase reaction buffer before addition of SDS-sample buffer and boiling for 5 min at 95 °C. Samples were analyzed by SDS-PAGE and immunoblotting. PP1CA-treated and untreated control samples were additionally analyzed by PRM. Experiments were performed as describe above except that following washing of the beads with co-IP buffer, proteins were prepared for LC-MS analysis including on-bead reduction, alkylation, tryptic digestion and TiO_2_-based enrichment of phosphopeptides using the EasyPhos method as described previously^86^.

### In vitro phosphatase assay

For the dephosphorylation of endogenous FLNc, lysates of differentiated C2 myotybes (1 mg of protein per sample) were mixed with 1 µM of recombinant PP1c^55^ in a total volume of 500 µL and incubated at 30 °C for the indicated period of time. The reaction was stopped by adding SDS sample buffer and immediate boiling for 5 min at 95 °C. Samples were analyzed by SDS-PAGE and immunoblotting.

### FILIP1 pulldown experiments

His-tagged FILIP1-2 cCT, recombinantly expressed in *E.coli* BL21(DE3), was coupled to Ni^2+^-NTA agarose beads (Qiagen, Hilden, Germany) as described before^26^ and washed once each with lysis buffer (50 mM NaH_2_PO_4_, 300 mM NaCl, 10 mM imidazole, pH 8) and washing buffer (same as lysis buffer containing 40 mM imidazole). FILIP1-2 cCT-coupled beads were mixed with cell lysates from mild EPS-treated C2 myotubes (1 mg of protein per replicate), which had been lysed in RIPA buffer supplemented with 2 mM MnCl_2_ and 1 mM DTT and incubated with 1 µM PP1CA or buffer as control (1 h at 30 °C). Samples were incubated end-over-end for 2 h at 8 °C. Supernatants were discarded, beads were washed twice with washing buffer and boiled (5 min, 95 °C) in SDS sample buffer. Samples were analyzed by SDS-PAGE and immunoblotting.

### LC-MS analysis

Reversed-phase LC-MS was performed using the UltiMate™ 3000 RSLCnano UHPLC system (Thermo Fisher Scientific, Dreieich, Germany) connected to a Q Exactive Plus mass spectrometer (Thermo Fisher Scientific, Bremen, Germany). The UHPLC system was equipped with two C18 pre-columns (µPAC™ trapping columns, PharmaFluidics/Thermo Fisher Scientific) and a C18 endcapped analytical column (50 cm µPAC™ column, PharmaFluidics/Thermo Fisher Scientific). Peptides were separated and eluted using a binary solvent system consisting of 0.1% formic acid (FA) (solvent A) and 80% ACN/0.1%FA (solvent B). Prior to LC-MS analysis, samples for PRM measurements were mixed with 95 fmol phosphopeptides of a phosphopeptide standard (Intavis). Peptide samples of all experiments were loaded onto the pre-column for 3 min at 1% solvent B and a flow rate of 10 µl/min. Peptides of PRM analyses were eluted with a gradient ranging from 1 to 20% solvent B in 22 min and 20 to 42% B in 11 min, followed by a 5-min flush at 95% B at a flow rate of 300 nl/min.

For the analysis of peptide mixtures obtained in hFlnc d18-21/d1-3 pulldown experiments, the gradient was as follows: 1–24% solvent B in 37 min, 24–50% B in 13 min, and a 5-min flush at 95% B (flow rate: 300 nl/min).

The Q Exactive Plus mass spectrometer was operated with a nano-electrospray ion source and distal coated SilicaTipemitter (New Objective, Littleton, USA) at a transfer capillary temperature of 250 °C and an ionization voltage of 1.6–1.7 kV. Tandem mass spectrometry (MS/MS) analyses of multiply charged peptide ions were performed in positive ion mode, using higher-energy collisional dissociation with a normalized collision energy of 28% for peptide fractionation.

For PRM analyses of hFLNc d18-21 from EPS-treated and control cells, an inclusion list containing 47 transitions was generated with Skyline (version 20.2.0.286). Each scan cycle consisted of a full MS1 scan with a resolution of 70,000, a scan range of *m/z* 375 to 1,700, an automatic gain control (AGC) target of 3E6 ions, and a maximum ion time (max. IT) of 60 ms, followed by 24 PRM scans with a resolution of 35,000, an AGC target of 1E5, a max. IT of 120 ms, and an isolation window of 1.8 *m/z*. For PRM analyses of PP1-treated hFLNc d18-21, the inclusion list contained 37 transitions. A scan cycle included a full MS1 scan (resolution of 70,000) with a range of *m/z* 400 to 1,600, an AGC target of 3E6 ions, and a max. IT of 20 ms, followed by 24 PRM scans with a resolution of 17,500, an AGC target of 2E5, a max. IT of 49 ms, and an isolation window of 2 *m/z*.

Acquisition of MS/MS data from samples of hFLNc d18-21/d1-3 pulldown experiment was performed applying a scan range of *m/z* 375 to 1,700, a resolution of 70,000, an AGC target of 3E6 ions, and a max. IT of 60 ms for MS survey scans. A TOP15 method was used for fragmentation of multiply charged precursor ions at a resolution of 35,000, an AGC target of 1E6, a max. IT of 120 ms, and a dynamic exclusion time of 45 s.

### MS data analysis and bioinformatics

All mass spectrometric raw data were processed using MaxQuant/Andromeda^89,90^ (version 1.6.10.43 for the analyses hFLNc d18-21 from EPS-treated and control cells and hFLNc d18-21/d1-3 pulldown experiments; version 2.2.0.0 for the analysis of PP1-treated hFLNc d18-21). Database searches were perfomed using the UniProt ProteomeSet human (including isoforms; version from 12/2019, 96,485 entries; hFLNc d18-21 from EPS-treated cells), the UniProt ProteomeSet mouse (03/2020; 63,722 entries), to which with the protein sequences of the FLNc constructs used for the experiment were added (hFLNc d18-21/d1-3 pulldown experiments), or the UniProt KB human (including isoforms; 02/2024, 103651 entries; PP1-treated hFLNc d18-21). Database searches were conducted using MaxQuant default settings, except that for the analysis of data from hFLNc d18-21/d1-3 pulldown experiments the minimum of unique peptides required for protein identification was set to 1. For the identification of phosphosites, phosphorylation of serine, threonine and tyrosine residues were set as variable modifications. Identified peptides were matched between runs to minimize the occurrence of missing values.

Targeted MS/MS data were analyzed using Skyline. MS intensities of hFLNc d18-21 phosphopeptides from EPS-treated and control cells were normalized replicate-wise to the mean intensity of selected phosphopeptides from the phosphopeptide standard. MS intensities of hFLNc d18-21 phosphopeptides from PP1-treated and control samples were normalized replicate-wise to the intensity of the non-phosphorylated FLNc variant. The significance of changes in phophopeptide abundance between different samples was calculated using a two-tailed Students t-test (*n* = 3). The results of the PRM analyses are provided in Supplementary Table 1 (phosphorylation of hFLNc d18-21 in EPS-treated and control cells) and Supplementary Table 3 (phosphorylation of PP1-treated hFLNc d18-21).

MS data obtained in hFLNc d18-21/d1-3 pulldown experiments were processed and analyzed using the Python module autoprot (version 0.2)^91^ (https://github.com/ag-warscheid/autoprot). MS intensities were normalized using mean shift and variance stabilization normalization^92^. For both hFLNc d18-21 and hFLNc d1-3 pulldown experiments, missing values were imputed for proteins identified in ≥ 4 out of 5 replicates using DIMA^93^ (https://github.com/kreutz-lab/DIMAR). For proteins identified in < 4 replicates, minimal imputation was performed by randomly drawing values from a downshifted, narrowed normal distribution generated from the non-missing entries applying a downshift of 2σ and a width of 0.3σ. For each protein identified in ≥ 4 replicates (i.e., prior to data imputation) of hFLNc d18-21 pulldown experiments, the log_2_-fold change of hFLNc d18-21 *versus*hFLNc d1-3 h was determined based on the MS intensities after normalization and imputation of missing values. Proteins significantly enriched with hFLNc d18-21 were identified using the “linear models for microarray data” (limma) approach^94,95^. P-values were corrected for multiple testing according to Benjamini-Hochberg^96^. Results of hFLNc d18-21/d1-3 pulldown experiments are provided in Supplementary Table 2.

### Material for peptide synthesis and purification

The 2-chlorotrityl resin were purchased from Novabiochem (Merck Millipore) and all used synthetic amino acids were from Novabiochem (Merck Millipore) and Carbosynth. All other synthetic reagents mentioned in the synthesis procedure below were obtained from Novabiochem, Sigma-Aldrich, or Carl Roth. Peptide synthesis was performed on a MultiPep RSi peptide synthesizer (Intavis Bioanalytical Instruments AG) using a separate syringe for each peptide in parallel. Peptides were purified using a 1260 Infinity II preparative HPLC system (Agilent Technologies) equipped with a VP 250/10 NUCLEODUR 100-5 C18 ec column (Macherey-Nagel) running a linear gradient of 10% acetonitrile (ACN; solvent A) to 70% ACN (solvent B), both with 0.05% TFA. Validation was carried out on a 1260 Infinity I/II HPLC System coupled to a 6120 Quadrupole ESI-MS (Agilent Technologies) using an EC 250/4 NUCLEODUR 100-5 C18 ec column (Macherey-Nagel) with a linear gradient from solvent A (10% ACN, 0.05% TFA) to solvent B (90% ACN, 0.05% TFA). Additionally, a Microflex LT matrix-assisted laser desportion/ionization (MALDI) system (Bruker) was employed to confirm correct peptide synthesis.

### Peptide synthesis and purification

Peptides were synthesized following previously reported base-mediated fluorenylmethyloxycarbonyl-(Fmoc) deprotection solid-phase peptide synthesis (SPPS) strategy^45,97,98^. 2-chlorotrityl resins pre-coupled with tert-butyloxycarbonyl–protected Glutamine were used for the synthesis of all peptides. Resins were swollen for 10 min in N, N-dimethylformamide (DMF) before synthesis in the syringe. One round of synthesis was performed for each (phospho-)amino acid of the peptide sequences. A synthetic round consisted of removal of the Fmoc group with piperidine followed by the subsequent coupling step of the following amino acid to the bound, side chain–protected amino acid residue, and is completed by a capping step with acetic anhydride (Ac_2_O). Coupling reactions were carried out by adding Fmoc-protected amino acids (4 eq), O-benzotriazole-N, N,N′,N′-tetramethyl-uronium hexafluorophosphate (HBTU) (4 eq), N-hydroxybenzotriazole hydrate (HOBt) (4 eq), and N-methylmorpholine (NMM, 4 eq) to the resin in 1 ml DMF and reacting for 30–45 min. Capping was achieved by adding 1 mL solution of 5% Ac_2_O and 5% 2,6-Lutidine in DMF for 5 min. Fmoc-deprotection was done by addition of a 20% piperidine solution (in DMF) to the resin, first for 3 min, then again for 8 min. Between each step resins were washed with DMF. After Fmoc deprotection of the last amino acid residue, peptides were cleaved from the resin and fully deprotected in one step by shaking overnight in cleavage cocktail (95% TFA, 2.5% triisopropylsilane (TIPS), and 2.5% H_2_O). Afterwards, peptides were precipitated from the acidic solution by adding cold diethylether (Et_2_O, − 20 °C) and collected by centrifugation (4000x*g*, at 4 °C, 5 min). Peptide precipitation was dried for 16 h under atmosphere, and then then validated by MALDI and analytical HPLC-MS and purified by Prep HPLC. The purified peptides were lyophilized and dissolved in 10% DMSO in water at a concentration of 10 mM for dephosphorylation assays.

### Peptide enzymatic assays

Purified peptides were dissolved in 10% DMSO/90% H_2_O at a final concentration of 10 mM. The EnzChek Phosphate Assay Kit (Thermo Fisher Scientific) was used to assess the kinetics/dynamics of peptide dephosphorylation by recombinant PP1c. The assay was carried out in a volume of 100 µL reaction buffer (20 mM Tris, 100 mM NaCl, 2 mM DTT, 0.15 U purine nucleoside phosphorylase, 0.2 mM 2-amino-6-mercapto-7-methylpurine riboside) containing indicated concentrations of peptide, and 25 nM recombinant PP1c. The change of absorbance was detected at 360 nm and 28 °C using a Synergy H1 microplate reader (BioTek). For analysis of enzyme kinetics, measured values were processed and compared to a standard curve prepared by using the phosphate solution included in the manufacturer’s kit and following the instructions. Data from three independent replicates were fitted to the Michaelis–Menten model and kinetic parameters from GraphPad Prism v6.0 (GraphPad Software Inc.) and extracted. Error bars represent SD of three replicates.

## Electronic supplementary material

Below is the link to the electronic supplementary material.

Supplementary Material 1

Supplementary Material 2

Supplementary Material 3

Supplementary Material 4

## Data Availability

Mass spectrometric raw data and MaxQuant result files have been deposited to the ProteomeXchange Consortium^99^via the PRIDE partner repository^100^ and are accessible using the dataset identifiers PXD053234 (FLNc-S2233/S2236 phosphorylation after EPS treatment), PXD053236 (Flnc d18-21/d1-3 pulldown experiments), and PXD53238 (hFLNc-S2233/S2236 phosphorylation after PP1 treatment).

## References

[1] Höhfeld, J. et al. Maintaining proteostasis under mechanical stress. *EMBO Rep.* **22**, e52507. 10.15252/embr.202152507 (2021). 34309183 10.15252/embr.202152507 PMC8339670

[2] Richter, K., Haslbeck, M. & Buchner, J. The heat shock response: life on the verge of death. *Mol. Cell*. **40**, 253–266. 10.1016/j.molcel.2010.10.006 (2010). 20965420 10.1016/j.molcel.2010.10.006

[3] Haze, K., Yoshida, H., Yanagi, H., Yura, T. & Mori, K. Mammalian transcription factor ATF6 is synthesized as a transmembrane protein and activated by proteolysis in response to endoplasmic reticulum stress. *Mol. Biol. Cell*. **10**, 3787–3799. 10.1091/mbc.10.11.3787 (1999). 10564271 10.1091/mbc.10.11.3787 PMC25679

[4] Hoshijima, M. Mechanical stress-strain sensors embedded in cardiac cytoskeleton: Z disk, titin, and associated structures. *Am. J. Physiol. Heart Circ. Physiol.* **290**10.1152/ajpheart.00816.2005 (2006). H1313-25. 10.1152/ajpheart.00816.2005 PMC3241960 16537787

[5] Frank, D., Kuhn, C., Katus, H. A. & Frey, N. Role of the sarcomeric Z-disc in the pathogenesis of cardiomyopathy. *Future Cardiol.* **3**, 611–622. 10.2217/14796678.3.6.611 (2007). 19804282 10.2217/14796678.3.6.611

[6] Frank, D. & Frey, N. Cardiac Z-disc signaling network. *J. Biol. Chem.* **286**, 9897–9904. 10.1074/jbc.R110.174268 (2011). 21257757 10.1074/jbc.R110.174268 PMC3060542

[7] Wadmore, K., Azad, A. J. & Gehmlich, K. The role of Z-disc proteins in Myopathy and Cardiomyopathy. *Int. J. Mol. Sci.* **22**10.3390/ijms22063058 (2021). 10.3390/ijms22063058 PMC8002584 33802723

[8] Razinia, Z., Mäkelä, T., Ylänne, J. & Calderwood, D. A. Filamins in mechanosensing and signaling. *Annual Rev. Biophys.* **41**, 227–246. 10.1146/annurev-biophys-050511-102252 (2012). 22404683 10.1146/annurev-biophys-050511-102252 PMC5508560

[9] Himmel, M., van der Ven, P. F. M., Stöcklein, W. & Fürst, D. O. The limits of promiscuity: isoform-specific dimerization of filamins. *Biochemistry*. **42**, 430–439. 10.1021/bi026501+ (2003). 12525170 10.1021/bi026501+

[10] Pudas, R., Kiema, T. R., Butler, P. J. G., Stewart, M. & Ylänne, J. Structural basis for vertebrate filamin dimerization. *Struct. (London England: 1993)*. **13**, 111–119. 10.1016/j.str.2004.10.014 (2005). 10.1016/j.str.2004.10.014 15642266

[11] Maestrini, E. et al. Mapping of two genes encoding isoforms of the actin binding protein ABP-280, a dystrophin like protein, to Xq28 and to chromosome 7. *Hum. Mol. Genet.* **2**, 761–766. 10.1093/hmg/2.6.761 (1993). 7689010 10.1093/hmg/2.6.761

[12] Dalkilic, I., Schienda, J., Thompson, T. G. & Kunkel, L. M. Loss of FilaminC (FLNc) results in severe defects in myogenesis and myotube structure. *Mol. Cell. Biol.* **26**, 6522–6534. 10.1128/MCB.00243-06 (2006). 16914736 10.1128/MCB.00243-06 PMC1592847

[13] Fürst, D. O. et al. Filamin C-related myopathies: pathology and mechanisms. *Acta Neuropathol.* **125**, 33–46. 10.1007/s00401-012-1054-9 (2013). 23109048 10.1007/s00401-012-1054-9 PMC5127197

[14] Brodehl, A. et al. Transgenic mice overexpressing desmocollin-2 (DSC2) develop cardiomyopathy associated with myocardial inflammation and fibrotic remodeling. *PloS One*. **12**, e0174019. 10.1371/journal.pone.0174019 (2017). 28339476 10.1371/journal.pone.0174019 PMC5365111

[15] Verdonschot, J. A. J. et al. A mutation update for the FLNC gene in myopathies and cardiomyopathies. *Hum. Mutat.* **41**, 1091–1111. 10.1002/humu.24004 (2020). 32112656 10.1002/humu.24004 PMC7318287

[16] Gigli, M. et al. Phenotypic expression, natural history, and risk stratification of Cardiomyopathy caused by Filamin C truncating variants. *Circulation*. **144**, 1600–1611. 10.1161/CIRCULATIONAHA.121.053521 (2021). 34587765 10.1161/CIRCULATIONAHA.121.053521 PMC8595845

[17] Eden, M. & Frey, N. Cardiac filaminopathies: Illuminating the Divergent Role of Filamin C mutations in human cardiomyopathy. *J. Clin. Med.* **10**10.3390/jcm10040577 (2021). 10.3390/jcm10040577 PMC7913873 33557094

[18] van der Ven, P. F. et al. Characterization of muscle filamin isoforms suggests a possible role of gamma-filamin/ABP-L in sarcomeric Z-disc formation. *Cell Motil. Cytoskeleton*. **45**, 149–162 (2000). 10.1002/(SICI)1097 – 0169(200002)45:2 < 149::AID-CM6 > 3.0.CO;2-G 10658210 10.1002/(SICI)1097-0169(200002)45:2<149::AID-CM6>3.0.CO;2-G

[19] Arndt, V. et al. Chaperone-assisted selective autophagy is essential for muscle maintenance. *Curr. Biology: CB*. **20**, 143–148. 10.1016/j.cub.2009.11.022 (2010). 20060297 10.1016/j.cub.2009.11.022

[20] Ulbricht, A. et al. Cellular mechanotransduction relies on tension-induced and chaperone-assisted autophagy. *Curr. Biology: CB*. **23**, 430–435. 10.1016/j.cub.2013.01.064 (2013). 23434281 10.1016/j.cub.2013.01.064

[21] Klimek, C., Kathage, B., Wördehoff, J. & Höhfeld, J. BAG3-mediated proteostasis at a glance. *J. Cell Sci.* **130**, 2781–2788. 10.1242/jcs.203679 (2017). 28808089 10.1242/jcs.203679

[22] Linnemann, A. et al. The sarcomeric Z-disc component myopodin is a multiadapter protein that interacts with filamin and alpha-actinin. *Eur. J. Cell Biol.* **89**, 681–692. 10.1016/j.ejcb.2010.04.004 (2010). 20554076 10.1016/j.ejcb.2010.04.004

[23] Collier, M. P. et al. HspB1 phosphorylation regulates its intramolecular dynamics and mechanosensitive molecular chaperone interaction with filamin C. *Sci. Adv.* **5**, eaav8421. 10.1126/sciadv.aav8421 (2019). 10.1126/sciadv.aav8421 PMC6530996 31131323

[24] van der Ven, P. F. M. et al. Unusual splicing events result in distinct Xin isoforms that associate differentially with filamin c and Mena/VASP. *Exp. Cell Res.* **312**, 2154–2167. 10.1016/j.yexcr.2006.03.015 (2006). 16631741 10.1016/j.yexcr.2006.03.015

[25] Lu, S., Carroll, S. L., Herrera, A. H., Ozanne, B. & Horowits, R. New N-RAP-binding partners alpha-actinin, filamin and Krp1 detected by yeast two-hybrid screening: implications for myofibril assembly. *J. Cell Sci.* **116**, 2169–2178. 10.1242/jcs.00425 (2003). 12692149 10.1242/jcs.00425

[26] Reimann, L. et al. Phosphoproteomics identifies dual-site phosphorylation in an extended basophilic motif regulating FILIP1-mediated degradation of filamin-C. *Commun. Biology*. **3**, 253. 10.1038/s42003-020-0982-5 (2020). 10.1038/s42003-020-0982-5 PMC7244511 32444788

[27] Reimann, L. et al. Myofibrillar Z-discs are a protein phosphorylation hot spot with protein kinase C (PKCα) modulating protein Dynamics. *Mol. Cell. Proteom.* **16**, 346–367. 10.1074/mcp.M116.065425 (2017). 10.1074/mcp.M116.065425 PMC5340999 28028127

[28] Murray, J. T., Campbell, D. G., Peggie, M., Mora, A. & Cohen, P. Identification of filamin C as a new physiological substrate of PKBalpha using KESTREL. *Biochem. J.* **384**, 489–494. 10.1042/bj20041058 (2004). 15461588 10.1042/BJ20041058 PMC1134134

[29] Nagano, T. et al. Filamin A-interacting protein (FILIP) regulates cortical cell migration out of the ventricular zone. *Nat. Cell Biol.* **4**, 495–501. 10.1038/ncb808 (2002). 12055638 10.1038/ncb808

[30] Orfanos, Z. et al. Breaking sarcomeres by in vitro exercise. *Sci. Rep.* **6**, 19614. 10.1038/srep19614 (2016). 26804343 10.1038/srep19614 PMC4726327

[31] Alessi, D. R. et al. Mechanism of activation of protein kinase B by insulin and IGF-1. *EMBO J.* **15**, 6541–6551 (1996). 8978681 PMC452479

[32] Sakamoto, K., Hirshman, M. F., Aschenbach, W. G. & Goodyear, L. J. Contraction regulation of akt in rat skeletal muscle. *J. Biol. Chem.* **277**, 11910–11917. 10.1074/jbc.M112410200 (2002). 11809761 10.1074/jbc.M112410200

[33] Swingle, M., Ni, L. & Honkanen, R. E. Small-molecule inhibitors of ser/thr protein phosphatases: specificity, use and common forms of abuse. *Methods Mol. Biology (Clifton N J)*. **365**, 23–38. 10.1385/1-59745-267-X:23 (2007). 10.1385/1-59745-267-X:23 PMC2709456 17200551

[34] Zhang, Q., Fan, Z., Zhang, L., You, Q. & Wang, L. Strategies for targeting Serine/Threonine protein phosphatases with small molecules in Cancer. *J. Med. Chem.* **64**, 8916–8938. 10.1021/acs.jmedchem.1c00631 (2021). 34156850 10.1021/acs.jmedchem.1c00631

[35] Toker, A. & Newton, A. C. Akt/protein kinase B is regulated by autophosphorylation at the hypothetical PDK-2 site. *J. Biol. Chem.* **275**, 8271–8274. 10.1074/jbc.275.12.8271 (2000). 10722653 10.1074/jbc.275.12.8271

[36] Xu, W. et al. The heat shock protein 90 inhibitor geldanamycin and the ErbB inhibitor ZD1839 promote rapid PP1 phosphatase-dependent inactivation of AKT in ErbB2 overexpressing breast cancer cells. *Cancer Res.* **63**, 7777–7784 (2003). 14633703

[37] Xiao, L. et al. Protein phosphatase-1 regulates Akt1 signal transduction pathway to control gene expression, cell survival and differentiation. *Cell Death Differ.* **17**, 1448–1462. 10.1038/cdd.2010.16 (2010). 20186153 10.1038/cdd.2010.16

[38] Cheng, Y. et al. MK-2206, a novel allosteric inhibitor of akt, synergizes with gefitinib against malignant glioma via modulating both autophagy and apoptosis Mol. *Cancer Ther* **11**, 154–164 (2012). 10.1158/1535-7163.MCT-11-0606 PMC3302182 22057914

[39] Ishihara, H. et al. Calyculin A and okadaic acid: inhibitors of protein phosphatase activity. *Biochem. Biophys. Res. Commun.* **159**, 871–877. 10.1016/0006-291X(89)92189-X (1989). 2539153 10.1016/0006-291x(89)92189-x

[40] Yamada, T. et al. 3-phosphoinositide-dependent protein kinase 1, an Akt1 kinase, is involved in dephosphorylation of Thr-308 of Akt1 in Chinese hamster ovary cells. *J. Biol. Chem.* **276**, 5339–5345. 10.1074/jbc.M005685200 (2001). 11087733 10.1074/jbc.M005685200

[41] Resjö, S. et al. Protein phosphatase 2A is the main phosphatase involved in the regulation of protein kinase B in rat adipocytes. *Cell. Signal.* **14**, 231–238. 10.1016/s0898-6568(01)00238-8 (2002). 11812651 10.1016/s0898-6568(01)00238-8

[42] Ugi, S. et al. Protein phosphatase 2A negatively regulates insulin’s metabolic signaling pathway by inhibiting akt (protein kinase B) activity in 3T3-L1 adipocytes. *Mol. Cell. Biol.* **24**, 8778–8789. 10.1128/MCB.24.19.8778-8789.2004 (2004). 15367694 10.1128/MCB.24.19.8778-8789.2004 PMC516764

[43] Bollen, M., Peti, W., Ragusa, M. J. & Beullens, M. The extended PP1 toolkit: designed to create specificity. *Trends Biochem. Sci.* **35**, 450–458 (2010). 20399103 10.1016/j.tibs.2010.03.002 PMC3131691

[44] Kokot, T. & Köhn, M. Emerging insights into serine/threonine-specific phosphoprotein phosphatase function and selectivity. *J. Cell Sci.* **135**10.1242/jcs.259618 (2022). 10.1242/jcs.259618 36205606

[45] Hoermann, B. et al. Dissecting the sequence determinants for dephosphorylation by the catalytic subunits of phosphatases PP1 and PP2A. *Nat. Commun.* **11**, 3583. 10.1038/s41467-020-17334-x (2020). 32681005 10.1038/s41467-020-17334-x PMC7367873

[46] Chatterjee, J. et al. Development of a peptide that selectively activates protein phosphatase-1 in living cells. *Angew. Chem. Int. Ed. Engl.* **51**, 10054–10059. 10.1002/anie.201204308 (2012). 22962028 10.1002/anie.201204308 PMC3531619

[47] Fischer, T. H. et al. Activation of protein phosphatase 1 by a selective phosphatase disrupting peptide reduces sarcoplasmic reticulum Ca2 + leak in human heart failure. *Eur. J. Heart Fail.* **20**, 1673–1685. 10.1002/ejhf.1297 (2018). 30191648 10.1002/ejhf.1297

[48] Wang, Y. et al. Interrogating PP1 activity in the MAPK pathway with optimized PP1-Disrupting peptides. *Chembiochem: Eur. J. Chem. Biology*. **20**, 66–71. 10.1002/cbic.201800541 (2019). 10.1002/cbic.201800541 PMC6471087 30338897

[49] Thayyullathil, F. et al. Protein phosphatase 1-dependent dephosphorylation of akt is the prime signaling event in sphingosine-induced apoptosis in Jurkat cells. *J. Cell. Biochem.* **112**, 1138–1153. 10.1002/jcb.23033 (2011). 21308747 10.1002/jcb.23033

[50] Tigges, U., Koch, B., Wissing, J., Jockusch, B. M. & Ziegler, W. H. The F-actin cross-linking and focal adhesion protein filamin A is a ligand and in vivo substrate for protein kinase C alpha. *J. Biol. Chem.* **278**, 23561–23569. 10.1074/jbc.M302302200 (2003). 12704190 10.1074/jbc.M302302200

[51] Hoffman, N. J. et al. Global phosphoproteomic analysis of human skeletal muscle reveals a network of Exercise-regulated kinases and AMPK substrates. *Cell Metabol.* **22**, 922–935. 10.1016/j.cmet.2015.09.001 (2015). 10.1016/j.cmet.2015.09.001 PMC4635038 26437602

[52] Rommel, C. et al. Differentiation stage-specific inhibition of the Raf-MEK-ERK pathway by Akt. *Sci. (New York N Y)*. **286**, 1738–1741. 10.1126/science.286.5445.1738 (1999). 10.1126/science.286.5445.1738 10576741

[53] Tong, J. F., Yan, X., Zhu, M. J. & Du, M. AMP-activated protein kinase enhances the expression of muscle-specific ubiquitin ligases despite its activation of IGF-1/Akt signaling in C2C12 myotubes. *J. Cell. Biochem.* **108**, 458–468. 10.1002/jcb.22272 (2009). 19639604 10.1002/jcb.22272

[54] Singh, S. et al. The reduced activity of PP-1α under redox stress condition is a consequence of GSH-mediated transient disulfide formation. *Sci. Rep.* **8**, 17711. 10.1038/s41598-018-36267-6 (2018). 30531830 10.1038/s41598-018-36267-6 PMC6286341

[55] Salvi, F. et al. Effects of stably incorporated iron on protein phosphatase-1 structure and activity. *FEBS Lett.* **592**, 4028–4038. 10.1002/1873-3468.13284 (2018). 30403291 10.1002/1873-3468.13284 PMC6587554

[56] Santos, C. X. C. et al. Targeted redox inhibition of protein phosphatase 1 by Nox4 regulates eIF2α-mediated stress signaling. *EMBO J.* **35**, 319–334. 10.15252/embj.201592394 (2016). 26742780 10.15252/embj.201592394 PMC4741303

[57] Heroes, E. et al. The PP1 binding code: a molecular-lego strategy that governs specificity. *FEBS J.* **280**, 584–595. 10.1111/j.1742-4658.2012.08547.x (2013). 22360570 10.1111/j.1742-4658.2012.08547.x

[58] van der Ven, P. F. et al. Indications for a novel muscular dystrophy pathway. gamma-filamin, the muscle-specific filamin isoform, interacts with myotilin. *J. Cell Biol.* **151**, 235–248. 10.1083/jcb.151.2.235 (2000). 11038172 10.1083/jcb.151.2.235 PMC2192634

[59] Yang, B. et al. Ectopic overexpression of filamin C scaffolds MEK1/2 and ERK1/2 to promote the progression of human hepatocellular carcinoma. *Cancer Lett.* **388**, 167–176. 10.1016/j.canlet.2016.11.037 (2017). 27919788 10.1016/j.canlet.2016.11.037

[60] Johnson, J. L. et al. A global atlas of substrate specificities for the human serine/threonine kinome (2022). 10.1038/s41586-022-05575-3 PMC9876800 36631611

[61] Hoermann, B. & Köhn, M. Evolutionary crossroads of cell signaling: PP1 and PP2A substrate sites in intrinsically disordered regions. *Biochem. Soc. Trans.* **49**, 1065–1074. 10.1042/BST20200175 (2021). 34100859 10.1042/BST20200175 PMC8286827

[62] Li, X., Wilmanns, M., Thornton, J. & Köhn, M. Elucidating human phosphatase-substrate networks. *Sci. Signal.* **6**, rs10. 10.1126/scisignal.2003203 (2013). 23674824 10.1126/scisignal.2003203

[63] Fedoryshchak, R. O. et al. Molecular basis for substrate specificity of the Phactr1/PP1 phosphatase holoenzyme. *eLife*. **9**10.7554/eLife.61509 (2020). 10.7554/eLife.61509 PMC7599070 32975518

[64] Köhn, M. Turn and face the strange: a New View on Phosphatases. *ACS Cent. Sci.* **6**, 467–477. 10.1021/acscentsci.9b00909 (2020). 32341996 10.1021/acscentsci.9b00909 PMC7181316

[65] Reddy, R. J. et al. Early signaling dynamics of the epidermal growth factor receptor. *Proc. Natl. Acad. Sci. U.S.A.* **113**, 3114–3119. 10.1073/pnas.1521288113 (2016). 26929352 10.1073/pnas.1521288113 PMC4801278

[66] Jiang, B. H., Aoki, M., Zheng, J. Z., Li, J. & Vogt, P. K. Myogenic signaling of phosphatidylinositol 3-kinase requires the serine-threonine kinase Akt/protein kinase B. *Proc. Natl. Acad. Sci. U.S.A.* **96**, 2077–2081. 10.1073/pnas.96.5.2077 (1999). 10051597 10.1073/pnas.96.5.2077 PMC26739

[67] Brunet, A. et al. Akt promotes cell survival by phosphorylating and inhibiting a forkhead transcription factor. *Cell*. **96**, 857–868. 10.1016/s0092-8674(00)80595-4 (1999). 10102273 10.1016/s0092-8674(00)80595-4

[68] Azad, A. J. et al. Functional analysis of a FLNC missense variant associated with hypertrophic cardiomyopathy. *J. Mol. Cell. Cardiol.* **173**10.1016/j.yjmcc.2022.08.033 (2022).

[69] Kley, R. A. et al. Impairment of protein degradation in myofibrillar myopathy caused by FLNC/filamin C mutations. *Autophagy*. **9**, 422–423. 10.4161/auto.22921 (2013). 23238331 10.4161/auto.22921 PMC3590265

[70] Kley, R. A. et al. FLNC-Associated Myofibrillar Myopathy: New Clinical, Functional, and Proteomic Data. *Neurol. Genet.* **7**, e590. 10.1212/NXG.0000000000000590 (2021). 34235269 10.1212/NXG.0000000000000590 PMC8237399

[71] Lad, Y. et al. Structure of three tandem filamin domains reveals auto-inhibition of ligand binding. *EMBO J.* **26**, 3993–4004. 10.1038/sj.emboj.7601827 (2007). 17690686 10.1038/sj.emboj.7601827 PMC1948075

[72] Rognoni, L., Stigler, J., Pelz, B., Ylänne, J. & Rief, M. Dynamic force sensing of filamin revealed in single-molecule experiments. *Proc. Natl. Acad. Sci. U.S.A.* **109**, 19679–19684. 10.1073/pnas.1211274109 (2012). 23150587 10.1073/pnas.1211274109 PMC3511698

[73] Vorgerd, M. et al. A mutation in the dimerization domain of filamin c causes a novel type of autosomal dominant myofibrillar myopathy. *Am. J. Hum. Genet.* **77**, 297–304. 10.1086/431959 (2005). 15929027 10.1086/431959 PMC1224531

[74] Kley, R. A. et al. Pathophysiology of protein aggregation and extended phenotyping in filaminopathy. *Brain: J. Neurol.* **135**, 2642–2660. 10.1093/brain/aws200 (2012). 10.1093/brain/aws200 PMC3437028 22961544

[75] Chevessier, F. et al. Myofibrillar instability exacerbated by acute exercise in filaminopathy. *Hum. Mol. Genet.* **24**, 7207–7220. 10.1093/hmg/ddv421 (2015). 26472074 10.1093/hmg/ddv421

[76] Schuld, J. et al. Homozygous expression of the myofibrillar myopathy-associated p.W2710X filamin C variant reveals major pathomechanisms of sarcomeric lesion formation. *Acta Neuropathol. Commun.* **8**10.1186/s40478-020-01001-9 (2020). 10.1186/s40478-020-01001-9 PMC7650280 32887649

[77] Leber, Y. et al. Filamin C is a highly dynamic protein associated with fast repair of myofibrillar microdamage. *Hum. Mol. Genet.* **25**, 2776–2788. 10.1093/hmg/ddw135 (2016). 27206985 10.1093/hmg/ddw135

[78] Eulitz, S. et al. Identification of Xin-repeat proteins as novel ligands of the SH3 domains of nebulin and nebulette and analysis of their interaction during myofibril formation and remodeling. *Mol. Biol. Cell*. **24**, 3215–3226. 10.1091/mbc.e13-04-0202 (2013). 23985323 10.1091/mbc.E13-04-0202 PMC3810769

[79] Molt, S. et al. Aciculin interacts with filamin C and Xin and is essential for myofibril assembly, remodeling and maintenance. *J. Cell Sci.* **127**, 3578–3592. 10.1242/jcs.152157 (2014). 24963132 10.1242/jcs.152157

[80] Nilsson, M. I. et al. Xin is a marker of skeletal muscle damage severity in myopathies. *Am. J. Pathol.* **183**, 1703–1709. 10.1016/j.ajpath.2013.08.010 (2013). 24225086 10.1016/j.ajpath.2013.08.010

[81] Grande, V., Schuld, J., van der Ven, P. F. M., Gruss, O. J. & Fürst, D. O. Filamin-A-interacting protein 1 (FILIP1) is a dual compartment protein linking myofibrils and microtubules during myogenic differentiation and upon mechanical stress. *Cell Tissue Res.* **393**, 133–147. 10.1007/s00441-023-03776-4 (2023). 37178194 10.1007/s00441-023-03776-4 PMC10313560

[82] Roos, A. et al. Bi-allelic variants of FILIP1 cause congenital myopathy, dysmorphism and neurological defects. *Brain: J. Neurol.* **146**, 4200–4216. 10.1093/brain/awad152 (2023). 10.1093/brain/awad152 PMC10545528 37163662

[83] Yaffe, D. & Saxel, O. Serial passaging and differentiation of myogenic cells isolated from dystrophic mouse muscle. *Nature*. **270**, 725–727. 10.1038/270725a0 (1977). 563524 10.1038/270725a0

[84] Gibson, D. G. et al. Enzymatic assembly of DNA molecules up to several hundred kilobases. *Nat. Methods*. **6**, 343–345. 10.1038/nmeth.1318 (2009). 19363495 10.1038/nmeth.1318

[85] Schindelin, J. et al. Fiji: an open-source platform for biological-image analysis. *Nat. Methods*. **9**, 676–682. 10.1038/nmeth.2019 (2012). 22743772 10.1038/nmeth.2019 PMC3855844

[86] Humphrey, S. J., Karayel, O., James, D. E. & Mann, M. High-throughput and high-sensitivity phosphoproteomics with the EasyPhos platform. *Nat. Protoc.* **13**, 1897–1916. 10.1038/s41596-018-0014-9 (2018). 30190555 10.1038/s41596-018-0014-9

[87] Peikert, C. D. et al. Charting organellar importomes by quantitative mass spectrometry. *Nat. Commun.* **8**, 15272. 10.1038/ncomms15272 (2017). 28485388 10.1038/ncomms15272 PMC5436138

[88] Rappsilber, J., Mann, M. & Ishihama, Y. Protocol for micro-purification, enrichment, pre-fractionation and storage of peptides for proteomics using StageTips. *Nat. Protoc.* **2**, 1896–1906. 10.1038/nprot.2007.261 (2007). 17703201 10.1038/nprot.2007.261

[89] Cox, J. & Mann, M. MaxQuant enables high peptide identification rates, individualized p.p.b.-range mass accuracies and proteome-wide protein quantification. *Nat. Biotechnol.* **26**, 1367–1372. 10.1038/nbt.1511 (2008). 19029910 10.1038/nbt.1511

[90] Cox, J. et al. Andromeda: a peptide search engine integrated into the MaxQuant environment. *J. Proteome Res.* **10**, 1794–1805. 10.1021/pr101065j (2011). 21254760 10.1021/pr101065j

[91] Bender, J., Mühlhäuser, W. W. D., Zimmerman, J. P., Drepper, F. & Warscheid, B. *Autoprot: Processing, Analysis and Visualization of Proteomics Data in Python* (2024).

[92] Huber, W., von Heydebreck, A., Sültmann, H., Poustka, A. & Vingron, M. Variance stabilization applied to microarray data calibration and to the quantification of differential expression. *Bioinf. (Oxford England)*. **18** (Suppl 1), 96–104. 10.1093/bioinformatics/18.suppl_1.s96 (2002). 10.1093/bioinformatics/18.suppl_1.s96 12169536

[93] Egert, J., Brombacher, E., Warscheid, B. & Kreutz, C. D. I. M. A. Data-Driven selection of an Imputation Algorithm. *J. Proteome Res.* **20**, 3489–3496. 10.1021/acs.jproteome.1c00119 (2021). 34062065 10.1021/acs.jproteome.1c00119

[94] Smyth, G. K. Linear models and empirical bayes methods for assessing differential expression in microarray experiments. *Stat. Appl. Genet. Mol. Biol.* **3**10.2202/1544-6115.1027 (2004). Article3. 10.2202/1544-6115.1027 16646809

[95] Schwämmle, V., León, I. R. & Jensen, O. N. Assessment and improvement of statistical tools for comparative proteomics analysis of sparse data sets with few experimental replicates. *J. Proteome Res.* **12**, 3874–3883. 10.1021/pr400045u (2013). 23875961 10.1021/pr400045u

[96] Benjamini, Y. & Hochberg, Y. Controlling the false Discovery rate: a practical and powerful Approach to multiple testing. *J. Roy. Stat. Soc.: Ser. B (Methodol.)*. **57**, 289–300. 10.1111/j.2517-6161.1995.tb02031.x (1995).

[97] Kokot, T. et al. PLDMS: Phosphopeptide Library Dephosphorylation followed by Mass Spectrometry Analysis to determine the specificity of Phosphatases for Dephosphorylation Site sequences. *Methods Mol. Biology (Clifton N J)*. **2499**, 43–64. 10.1007/978-1-0716-2317-6_2 (2022). 10.1007/978-1-0716-2317-6_2 35696074

[98] Schwarz, J. J. et al. Quantitative proteomics identifies PTP1B as modulator of B cell antigen receptor signaling. *Life Sci. Alliance*. **4**10.26508/lsa.202101084 (2021). 10.26508/lsa.202101084 PMC8473724 34526379

[99] Deutsch, E. W. et al. The ProteomeXchange consortium at 10 years: 2023 update. *Nucleic Acids Res.* **51**10.1093/nar/gkac1040 (2023). 10.1093/nar/gkac1040 PMC9825490 36370099

[100] Perez-Riverol, Y. et al. The PRIDE database resources in 2022: a hub for mass spectrometry-based proteomics evidences. *Nucleic Acids Res.* **50**10.1093/nar/gkab1038 (2022). D543-D552. 10.1093/nar/gkab1038 PMC8728295 34723319

[101] Pino, L. K. et al. The Skyline ecosystem: Informatics for quantitative mass spectrometry proteomics. *Mass Spectrom. Rev.* **39**, 229–244. 10.1002/mas.21540 (2020). 28691345 10.1002/mas.21540 PMC5799042

