# Supplementary material for: Protein phosphatase-1 regulates the binding of filamin C to FILIP1 in cultured skeletal muscle cells under mechanical stress
Source: Sci Rep. 2024 Nov 9;14:27348. doi: 10.1038/s41598-024-78953-8 (PMC11550807; doi:10.1038/s41598-024-78953-8)
Supplement: Supplementary file 3 — Supplementary Material 3 [file 41598_2024_78953_MOESM3_ESM.pdf]

SUPPLEMENTARY INFORMATIONS

Protein phosphatase-1 regulates the binding of Filamin C to FILIP1 in cultured skeletal muscle cells under mechanical stress

Thomas Kokot, Johannes P. Zimmermann, Anja N. Schwäble, Lena Reimann, Anna L. Herr, Nico Höfflin, Maja Köhn, Bettina Warscheid

SUPPLEMENTARY FIGURES

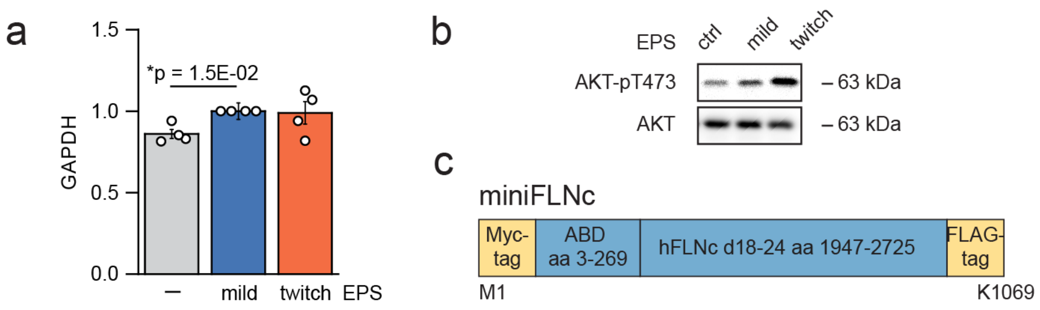

Supplementary Figure 1 (related to Figure 1): (a) Quantification of GAPDH signals in the immunoblots normalized to the mild EPS treatment. GAPDH was used as a general loading control and quantification showed that levels were equal between mild and twitch EPS condition. A two-tailed, paired student's t-test was performed, and data is presented as bar chart (n= 4). **(b)** Western blot showing AKT-pS473 as readout for AKT activity and total AKT levels in C2 myotubes under the EPS conditions shown in Figure 1a. **(c)** Illustration of the hFLNc d18-24 construct expressed in C2 myotubes, flanked by Myc-tag at the *N*-terminus and a Flag-tag at the *C*-terminus.

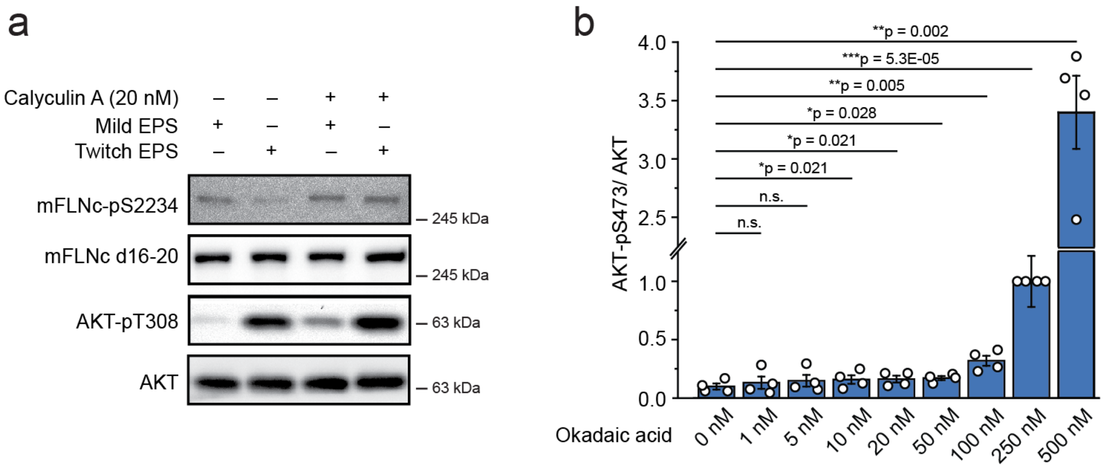

Supplementary Figure 2 (related to Figure 2): Phosphoprotein phosphatase inhibition results in increased levels of AKT-T308/S473 and mFLNc-S2234 phosphorylation. (a) Western blot analysis of calyculin A-treated C2 myotubes under mild and twitch EPS using total and phosphosite-specific antibodies as indicated. **(b)** Quantitative data of AKT-pS473 levels obtained from Western blot analysis of okadaic acid-treated C2 myotubes under EPS- (representative blots are shown in Figure 2d). A significant increase in AKT-pS473 levels was observed at a minimum okadaic acid concentration of 10 nM. Phospho-specific signal intensities of AKT-pS473 were normalized to the respective total AKT signal intensities. A two-tailed, paired student's t-test was performed and error bars represent the SEM (n=4).

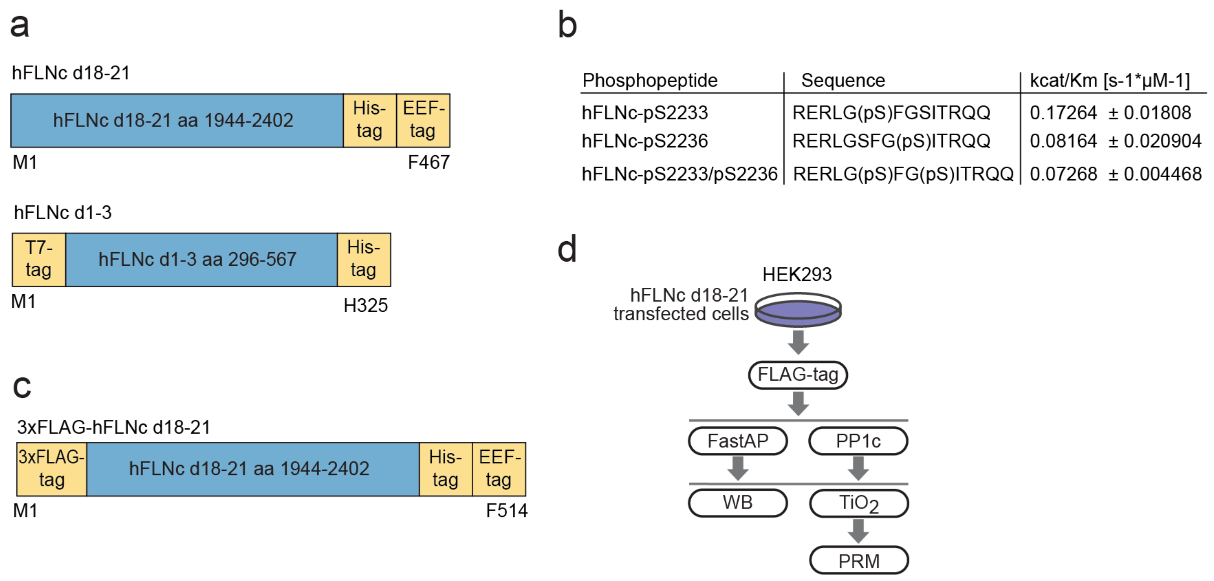

Supplementary Figure 3 (related to Figure 3): (a) Schematic illustration of the human (h) FLNc constructs used for pulldown analysis. **(b)** Sequences of synthesized phosphopeptides mimicking the hFLNc 2229-2242 sequence with the calculated k_{cat}/K_m values upon PP1 dephosphorylation. **(c)** Depiction of 3xFlag-tagged hFLNc d18-21 construct used for HEK293 transfection and subsequent on-bead dephosphorylation as illustrated in (d). Same construct was used for FILIP pulldown experiments shown in Figure 5a. **(d)** Illustrative workflow for the analysis of hFLNc d18-21 dephosphorylation by protein phosphatase 1c (PP1c) or fast alkaline phosphatase (Fast AP) after anti-FLAG immunopurification from HEK293 cell lysates. Dephosphorylation was monitored by Western blot (WB) analysis or parallel reaction monitoring (PRM) following phosphopeptide enrichment using TiO_2 beads.

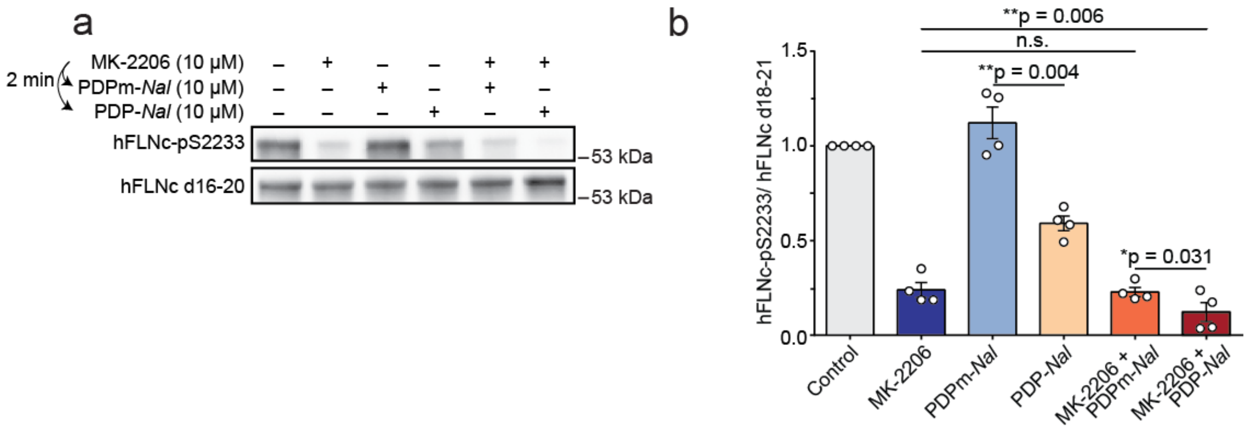

Supplementary Figure 4 (related to Figure 4): Activation of PP1 through PDP-Nal results in dephosphorylation of hFLNc-pS2233 in HEK293 cells. (a) HEK293 were treated with combinations of the AKT inhibitor MK-2206 and the PP1 activator peptide PDP-*Nal* or the respective control PDPm-*Nal* as indicated. Flag-tagged recombinant hFLNc (**Supplementary Figure 3c**) was captured through anti-FLAG immunoprecipitation and analyzed by Western blotting. Total and phospho-specific antibodies were used to display hFLNc-pS2233 levels and total hFLNc. **(b)** Quantification of immunoblot data exemplarily shown in (a). Phospho-specific signal intensities were normalized to the respective total signal intensities. A two-tailed, paired student's t-test was carried out, error bars represent the SEM (n=4).

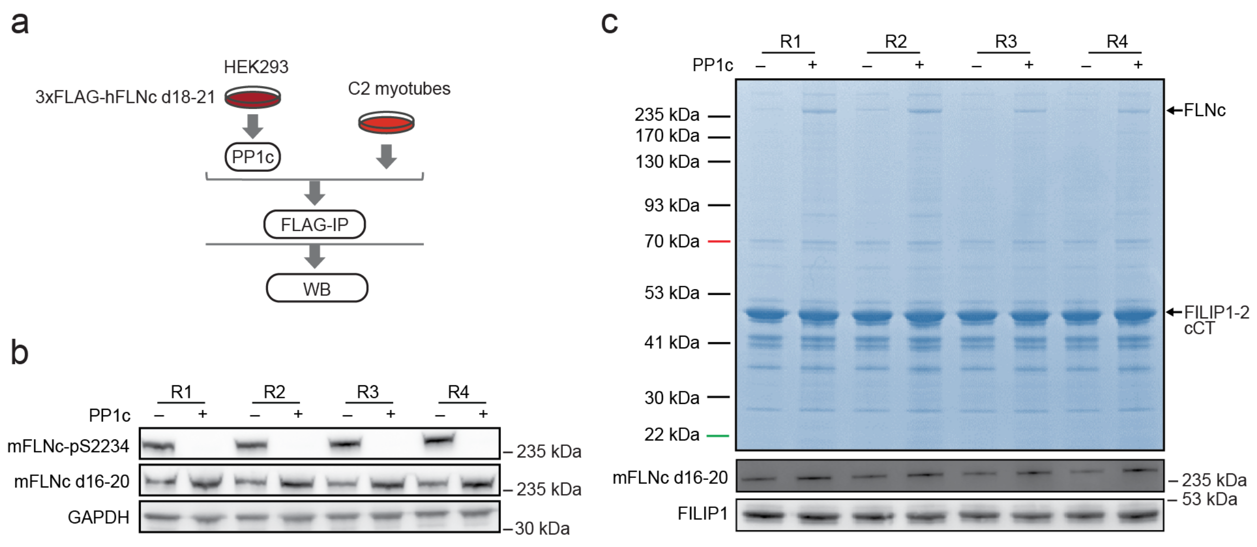

Supplementary Figure 5 (related to Figure 5): PP1 dephosphorylates endogenous mFLNc-pS2234 resulting in increased FILIP1 binding. (a) Workflow for the analysis of differences in the binding of endogenous FILIP1 to hFLNc d18-21 in dependency of PP1c-catalyzed PP1c. HEK293 cells were transfected with 3xFlag-tagged hFLNc d18-21 and lysates were treated with recombinant PP1 followed by immunoprecipitation using anti-Flag beads. Subsequently, pre-coupled hFLNc is incubated with C2 myotube lysate and eluates were analyzed by Western blotting. **(b)** Western blot analysis of C2 myotube lysate incubated with recombinant PP1c to verify PP1c-catalzyed dephosphorylation of endogenous mFLNc prior to the pulldown using His-tagged FILIP1-2 cCT coupled to Ni-NTA agarose beads. **(c)** SDS-PAGE and immunoblot analysis of the Ni-NTA-pulldown experiment to analyze changes in the binding of endogenous mFLNc to FILIP1 in dependency of PP1c-catalazyed dephosphorylation of mFLNc-pS2234.

SUPPLEMENTARY TABLES

Supplementary Table 1 (related to Figure 1c): Phosphorylation of hFLNc at S2233 and S2236 in EPS-treated and untreated C2 myotubes. FLNc phosphopeptides were enriched using TiO_2 beads, and tryptic peptides, mixed with a phosphopeptide standard, were analyzed by targeted LC-MS using parallel reaction monitoring. MS data in the table were extracted from the MS raw files using Skyline (version 20.2.0.286). Raw MS data and MaxQuant results files are available via ProteomeXchange with the identifier PXD053234.

Supplementary Table 2 (related to Figure 3a): Identification of FLNc d18-21 interaction partners by quantitative label-free mass spectrometry. Raw MS data and MaxQuant results files are available via ProteomeXchange with the identifier PXD053236.

Supplementary Table 3 (related to Figure 3d): Dephosphorylation of hFLNc-pS2233 by PP1c *in vitro*. FLNc phosphopeptides were enriched using TiO_2 beads, and tryptic peptides, mixed with a phosphopeptide standard, were analyzed by targeted LC-MS using parallel reaction monitoring. MS data in the table were extracted from the MS raw files using Skyline (version 20.2.0.286). Raw MS data and MaxQuant results files are available via ProteomeXchange with the identifier PXD053238.

FULL-LENGTH GELS AND PLOTS

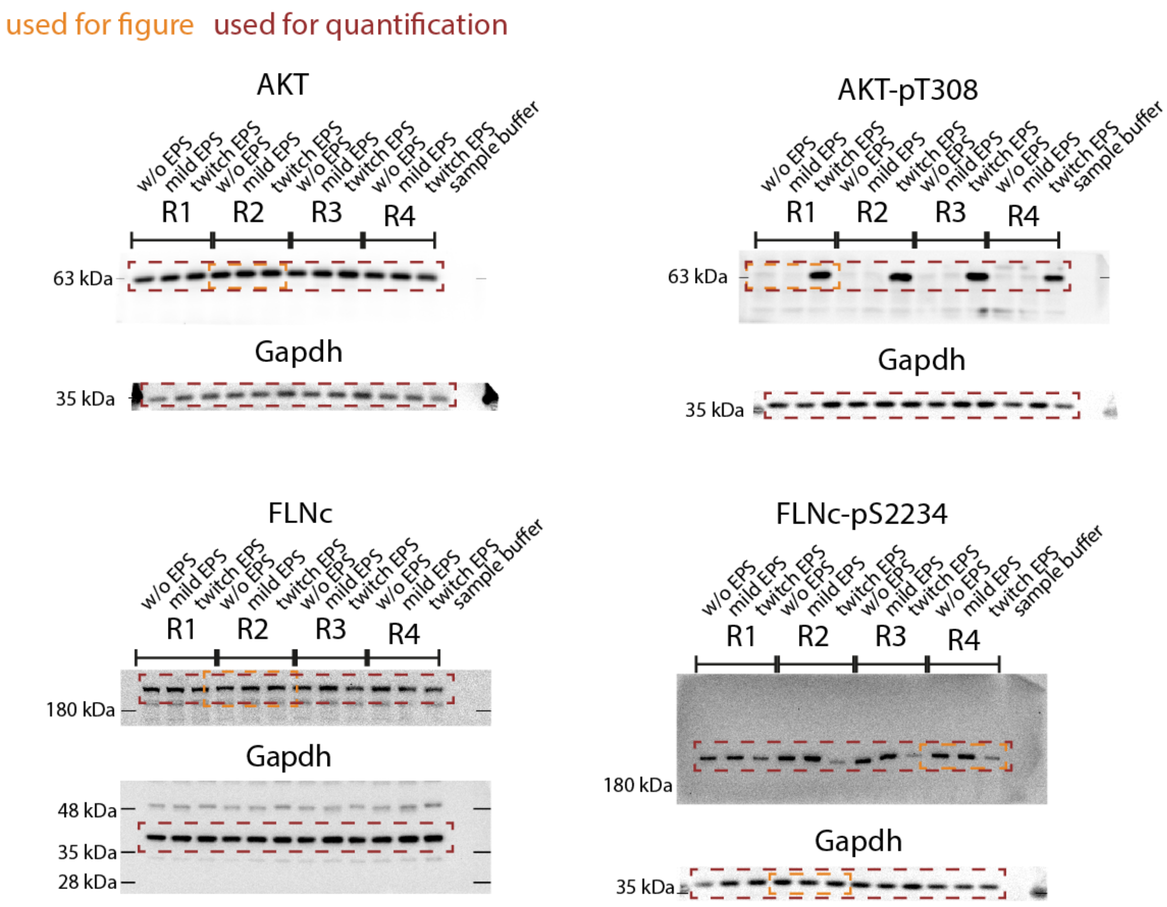

Uncropped Blots Figure 1a, Supplementary Figure 1b:

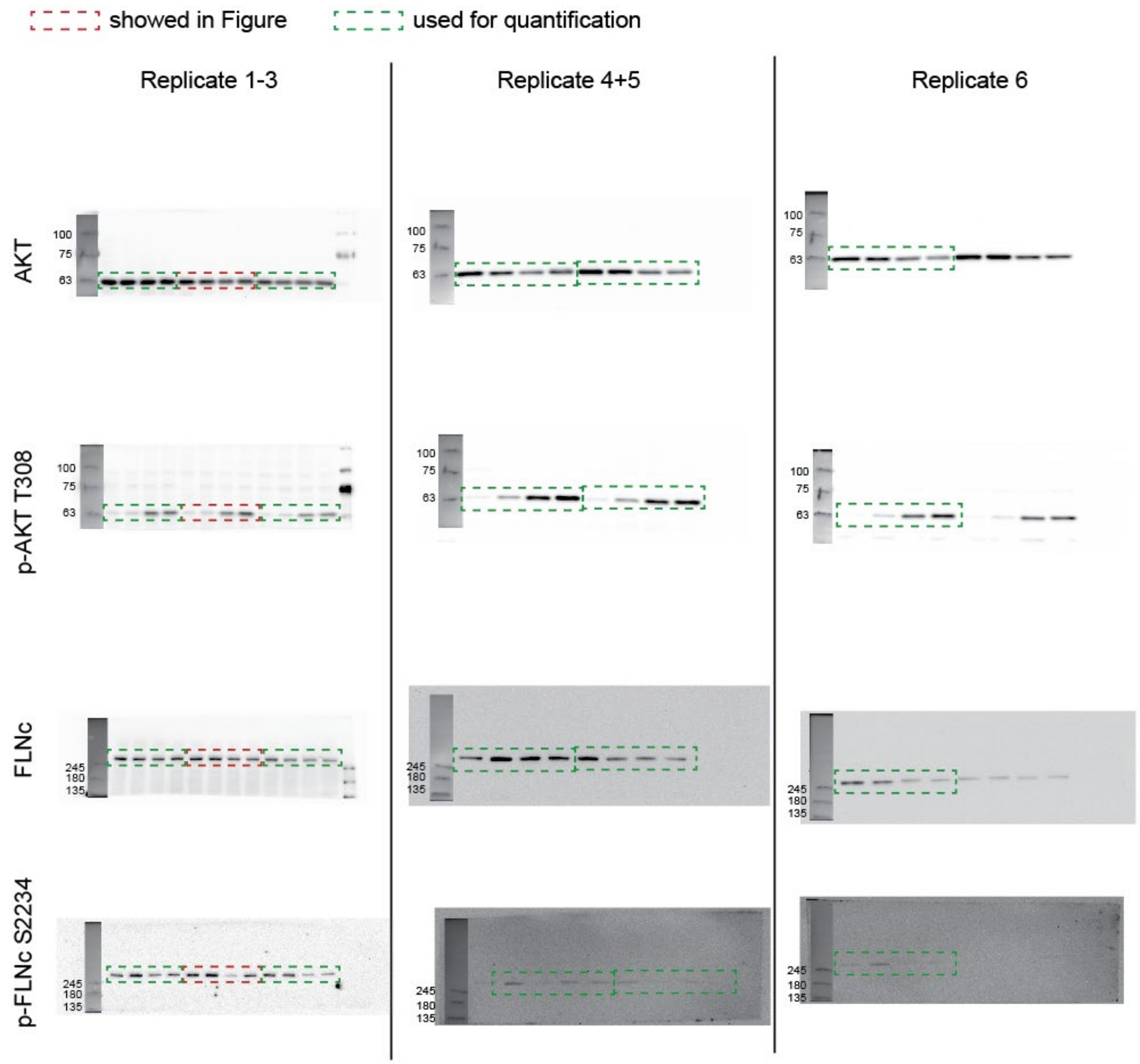

Uncropped Blots Figure 2a:

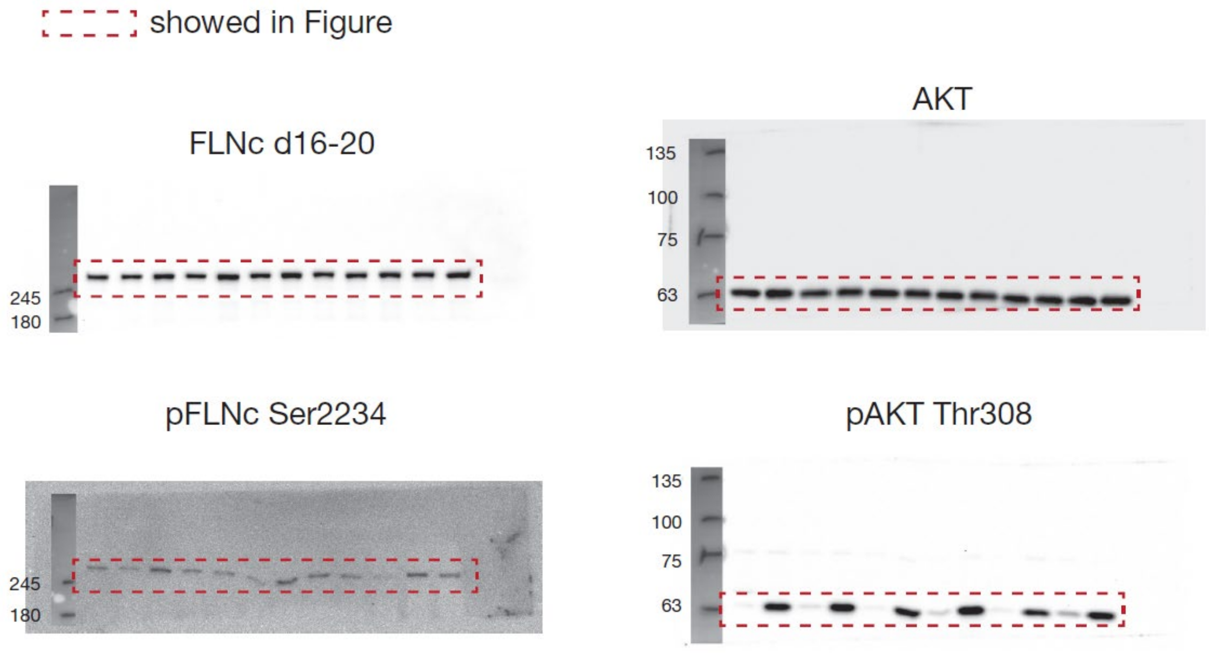

Uncropped Blots Supplementary Figure 2a:

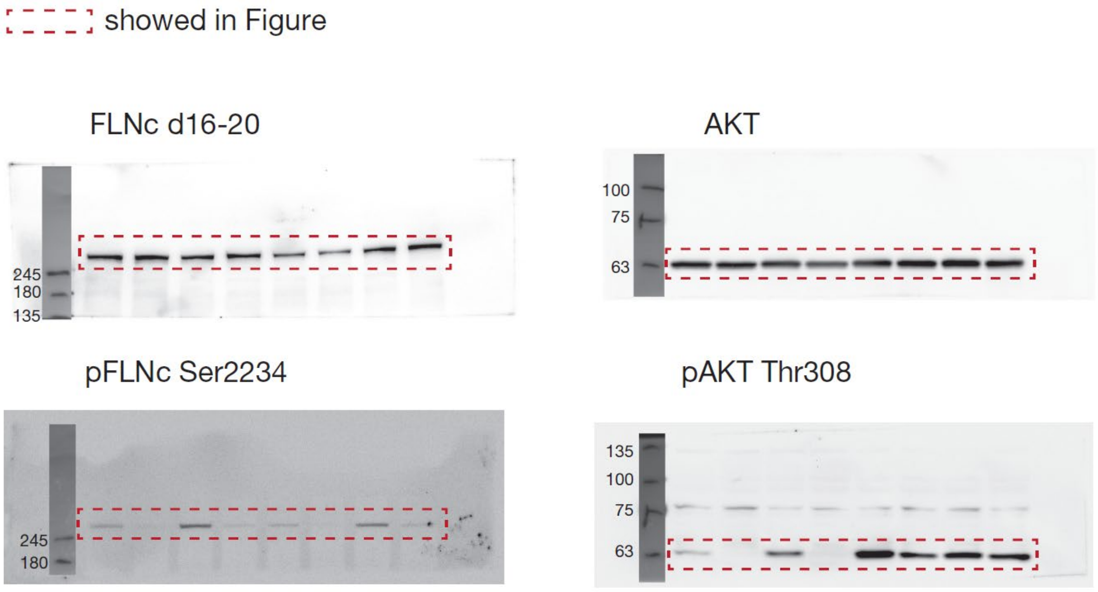

Uncropped Blots Figure 2d:

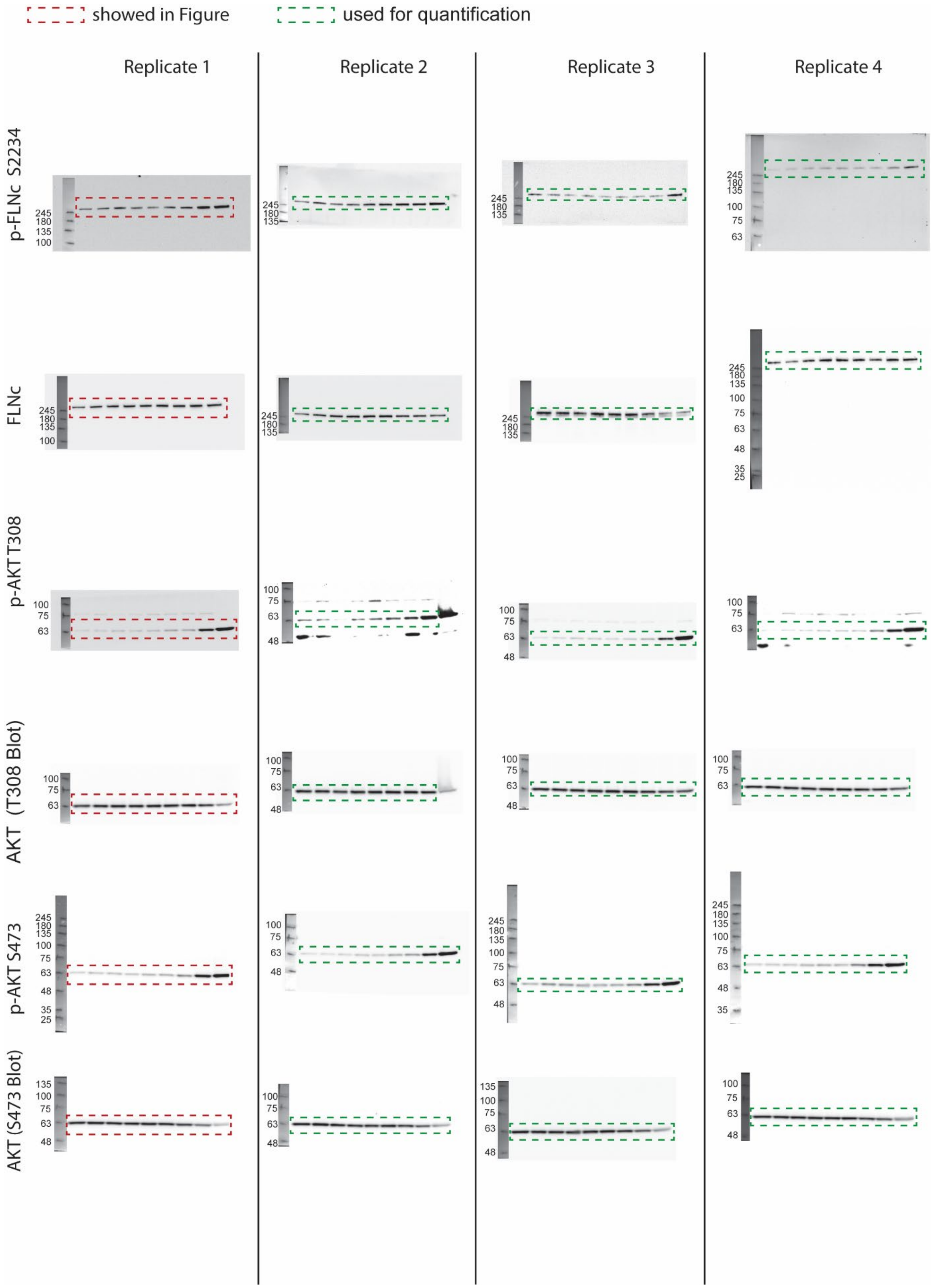

Uncropped Blots Figure 2e:

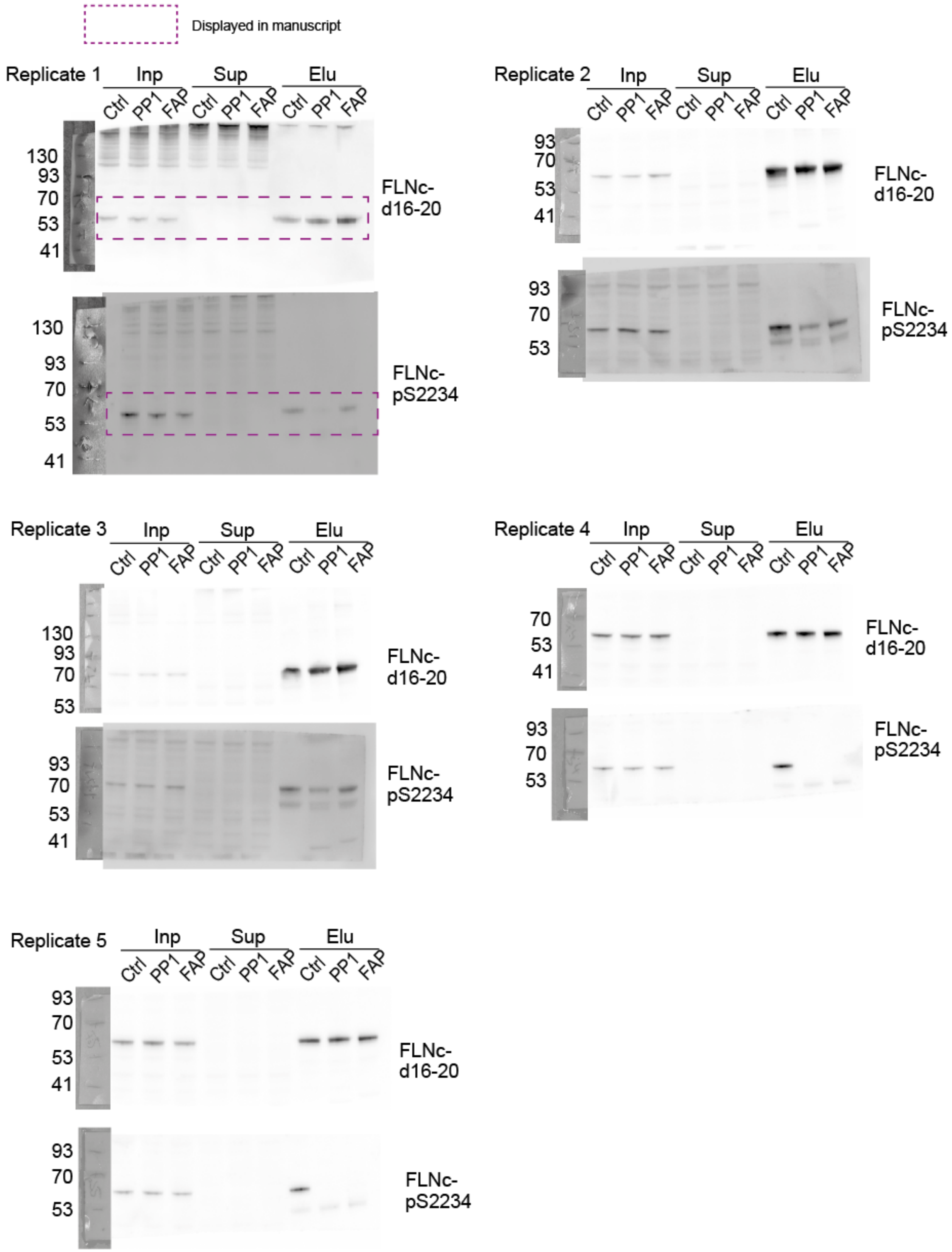

Uncropped Blots Figure 3c:

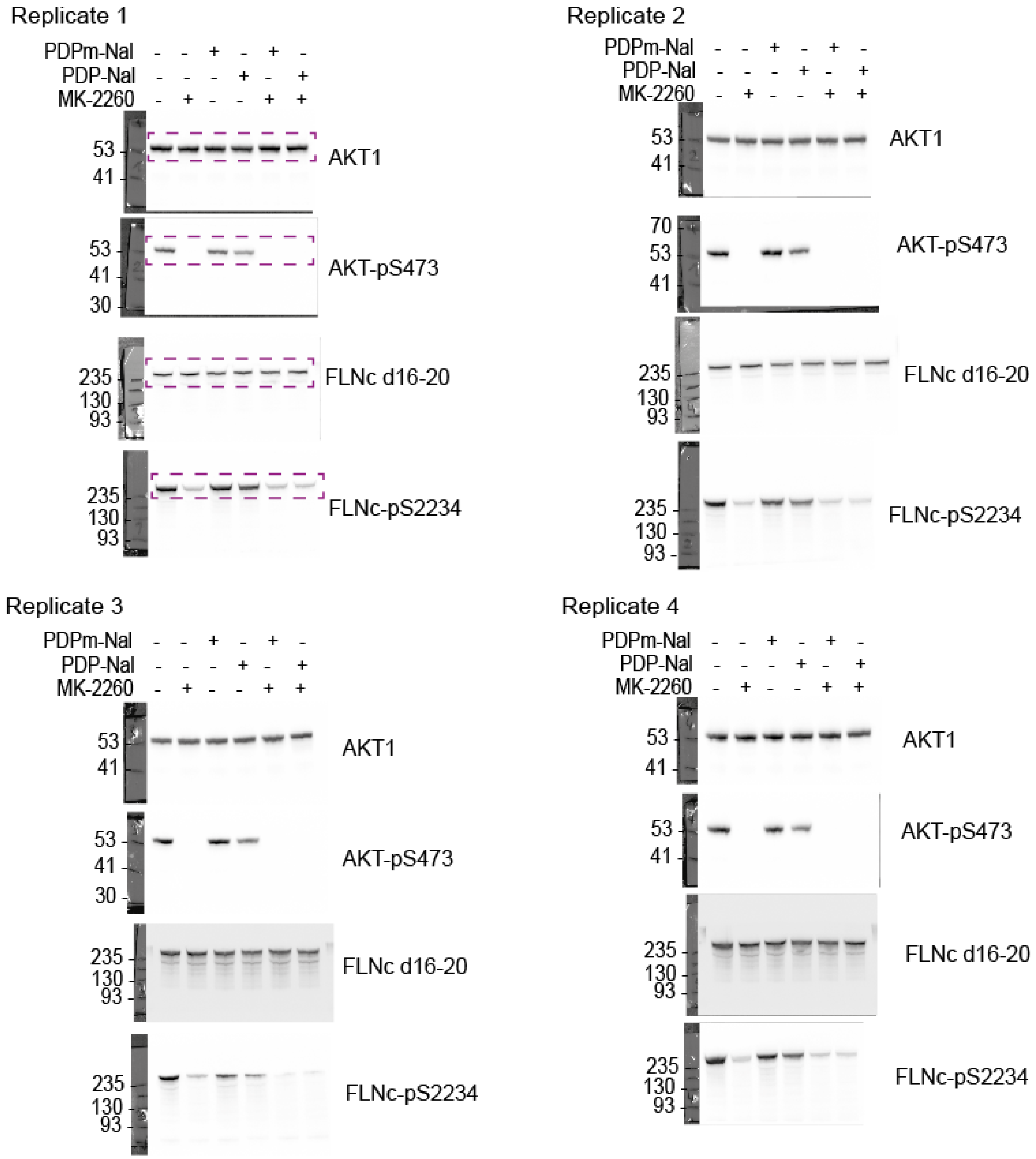

Uncropped Blots Figure 4a:

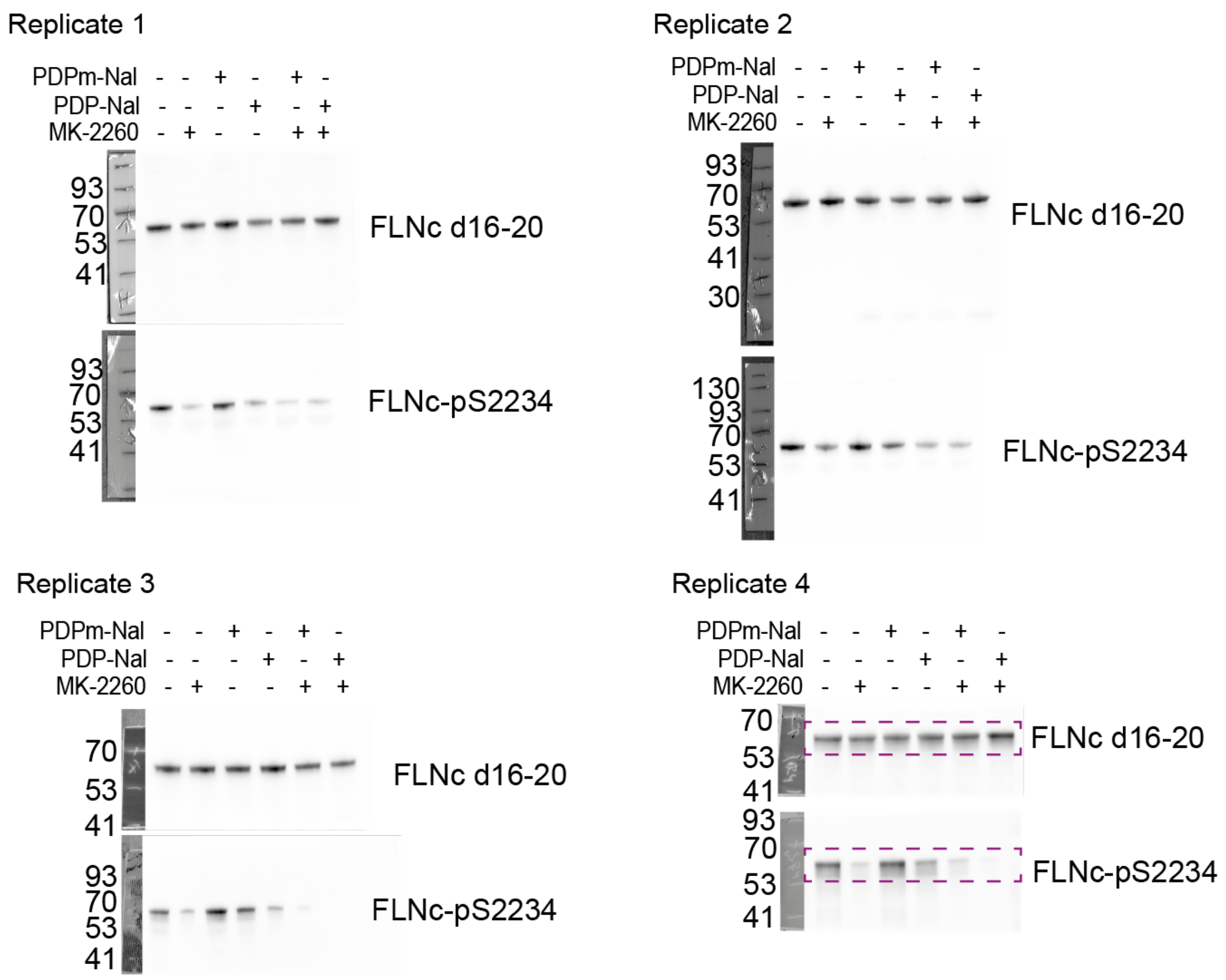

Uncropped Blots Supplementary Figure 4a:

Replicate 1

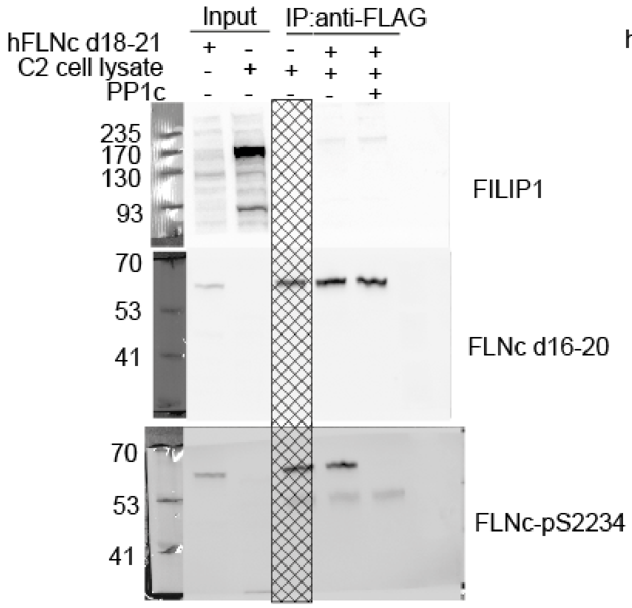

Replicate 2 - Replicate 4

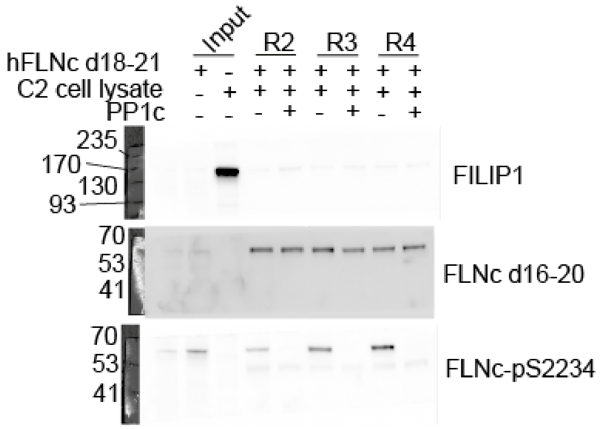

Replicate 5

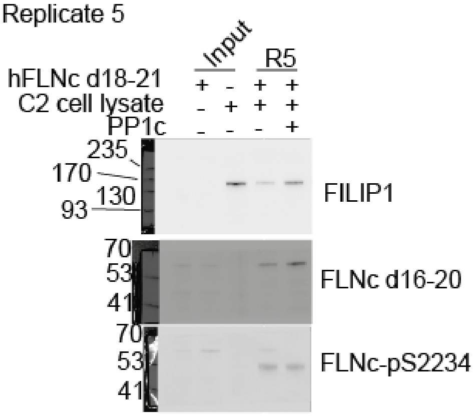

Replicate 6

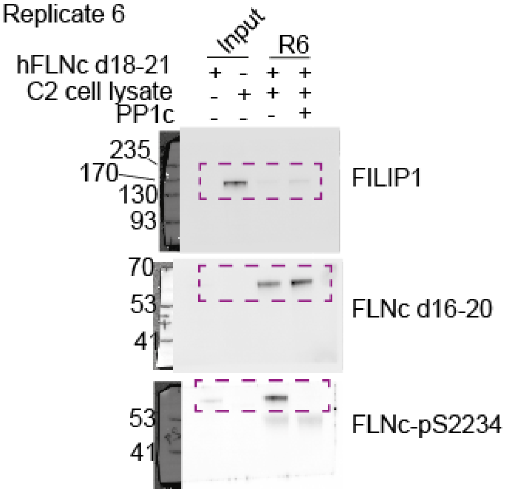

Uncropped Blots Figure 5a:

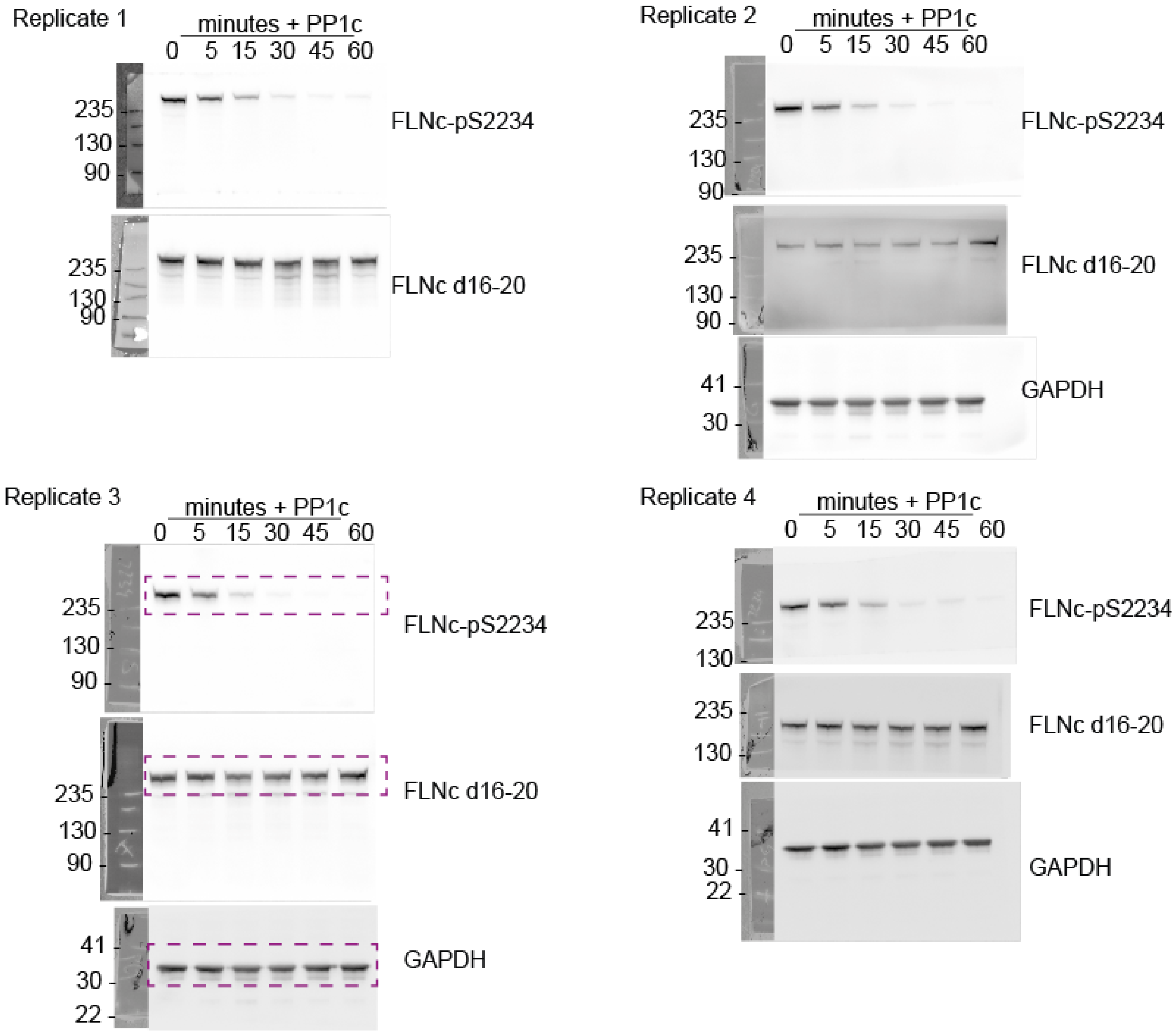
Replicate 1
minutes + PP1c
0 5 15 30 45 60
235
130
90
FLNc-pS2234
235
130
90
FLNc d16-20
Replicate 2
minutes + PP1c
0 5 15 30 45 60
235
130
90
FLNc-pS2234
235
130
90
FLNc d16-20
41
30
GAPDH
Replicate 3
minutes + PP1c
0 5 15 30 45 60
235
130
90
FLNc-pS2234
235
130
90
FLNc d16-20
41
30
22
GAPDH
Replicate 4
minutes + PP1c
0 5 15 30 45 60
235
130
FLNc-pS2234
235
130
FLNc d16-20
41
30
22
GAPDH

Ctrl -R1

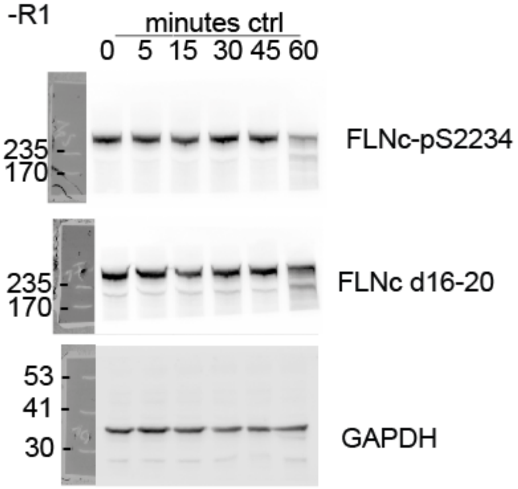

Ctrl -R2

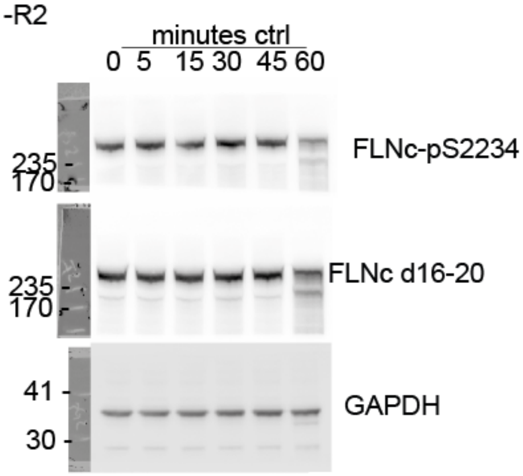

Ctrl-R3

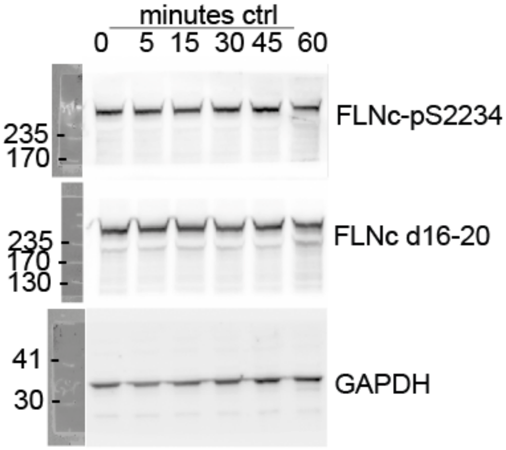

Ctrl-R4

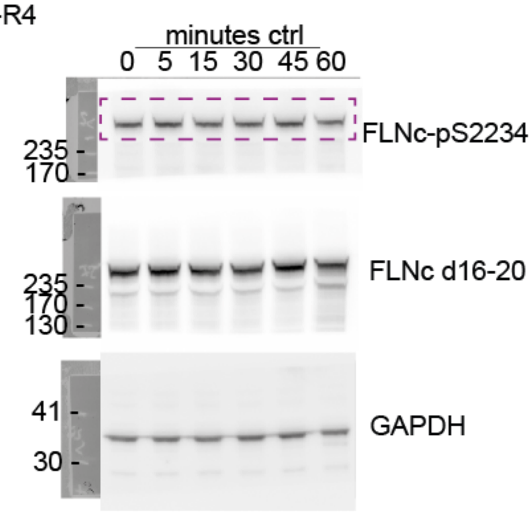

Uncropped Blots Figure 5c:

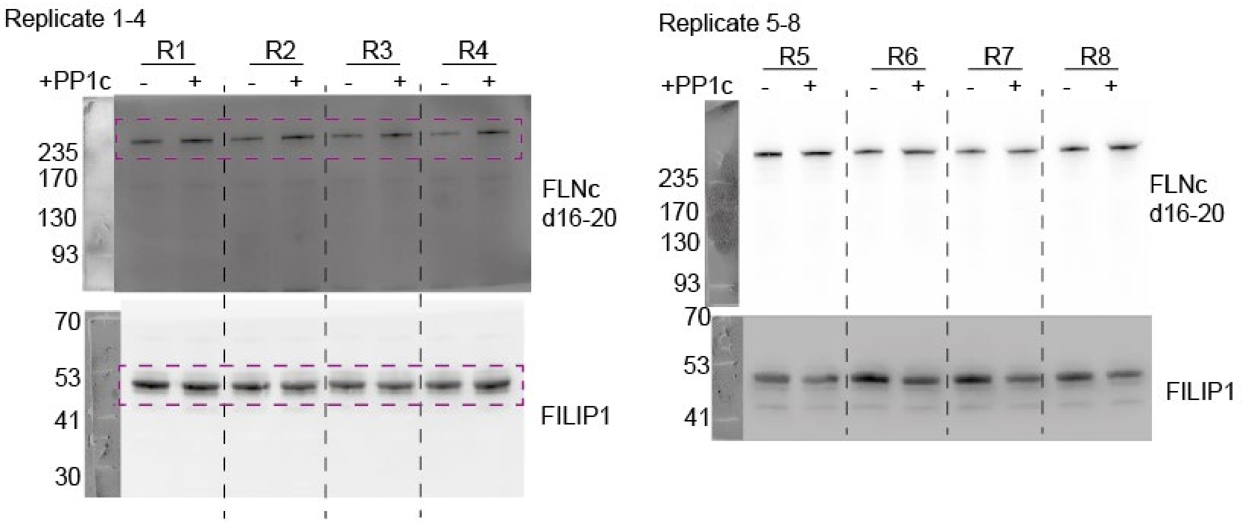

Uncropped Blots Figure 5f, Supplementary Figure 5c:

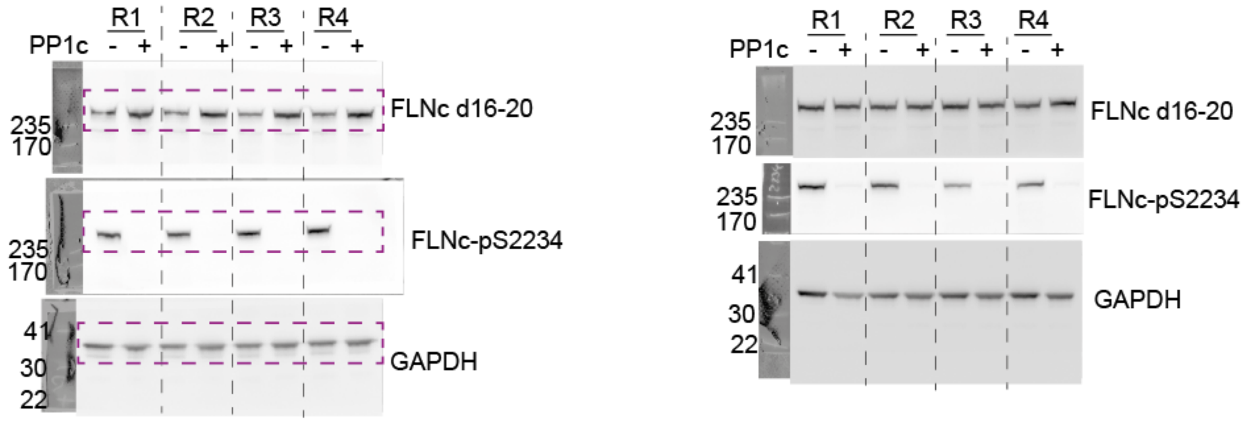

Uncropped Blots Supplementary Figure 5b:
